# Disruption to TFEB signaling and autophagy in newly formed oligodendrocytes leads to aberrant generation of CNS myelin

**DOI:** 10.1073/pnas.2528668123

**Published:** 2026-06-10

**Authors:** Daniela Barbosa, Aksheev Bhambri, Miguel Vasquez, Yihe Zhang, Gabrielle Sanchez, Katherine J. Wert, Natalia V Gounko, Mark H. Ellisman, Lu O. Sun

**Affiliations:** ^a^ Department of Molecular Biology, University of Texas Southwestern Medical Center, Dallas, TX 75390; ^b^ National Center for Microscopy and Imaging Research, Department of Neuroscience, University of California San Diego, La Jolla, CA 92039; ^c^ Department of Ophthalmology, University of Texas Southwestern Medical Center, Dallas, TX 75390; ^d^ Hamon Center for Regenerative Science and Medicine, University of Texas Southwestern Medical Center, Dallas, TX 75390; ^e^ Peter O’Donnell Jr. Brain Institute, University of Texas Southwestern Medical Center, Dallas, TX 75390; ^f^ Electron Microscopy Core Facility, Department of Cell Biology, University of Texas Southwestern Medical Center, Dallas, TX 75390

**Keywords:** axon ensheathment, myelin integrity, volume electron microscopy, newly formed oligodendrocytes

## Abstract

Myelin is a defining feature of the vertebrate nervous system, yet the cellular and molecular mechanisms governing its integrity remain poorly understood. Here, using volume electron microscopy and a knock-in mouse line targeting newly formed oligodendrocytes, we reconstruct early optic nerve myelination and examine retinal ganglion cell axon ensheathment. We observe that newly formed myelin sheaths exhibit membrane protrusions and occasional degenerative myelin “whorls.” Conditional disruption of the transcription factor EB (TFEB)-autophagy pathway in newly formed oligodendrocytes significantly increases the abundance of these aberrant myelin structures, indicating that this pathway is required for proper myelin formation and integrity. Importantly, this pathway acts independently of the well-established function of TFEB that represses myelin sheath growth. Together, our findings identify a role for TFEB-dependent autophagy in establishing proper myelin structure during development, providing insights into the oligodendrocyte-intrinsic mechanisms that regulate myelin integrity.

## Significance

The cellular and molecular mechanisms governing myelin integrity during axon ensheathment remain poorly understood. Using volume electron microscopy and a knock-in mouse line labeling newly formed oligodendrocytes and their myelin sheaths, we show that the transcription factor EB–autophagy pathway is required for proper myelin formation during axon ensheathment. While membrane protrusions and myelin whorls are observed at low frequencies under physiological conditions, disruption of this pathway in newly formed oligodendrocytes markedly increases these aberrant structures, compromising myelin integrity. Our work addresses an important question in the establishment of myelination, providing insights into the oligodendrocyte-intrinsic mechanisms that regulate myelin integrity and may be relevant to demyelinating diseases.

In the central nervous system (CNS), oligodendrocytes generate myelin sheaths that are assembled from large areas of plasma membrane (1). Myelin sheaths provide critical insulation and trophic support for axons, ensuring proper nervous system function (2). A major portion of the sheath is formed within a short period of time, a process known as axon ensheathment, to provide maximum myelin coverage for axons at the onset of myelination (3). Due to their large surface area, myelin sheaths comprise approximately 50% of the total dry mass of the human brain (1). Their extensive area makes them particularly vulnerable to environmental toxins and activated immune cells (1).

Recent work shows that myelin abnormalities, such as myelin whorls, outfoldings, and myelin-on-myelin structures, arise during development and gradually resolve over time (4). Intriguingly, similar myelin abnormalities reappear in mouse models of demyelination and in the early stages of multiple sclerosis (5–7). Among them, degenerative myelin whorls or myelinosomes, characterized by multilayered degenerative myelin, are a major myelin abnormality frequently observed in human demyelinating diseases prior to catastrophic demyelination, such as oligodendrocyte process degeneration and cell loss (7). Myelin whorls arise from excessive myelin growth, aberrant myelin compaction, disrupted actin cytoskeletal dynamics, or malformation of the node of Ranvier (8–12). For instance, genetic deletion of *Cnp1*, a critical gene responsible for myelin outgrowth and compaction, significantly increases whorl density during development (8, 9). In addition, disruption of actin polymerization in *N-Wasp* conditional knockout mice leads to increased myelin whorls and outfoldings (10). Last, myelin whorls appear along with double myelin and myelin outfoldings in *Cntn1^−/−^; Mag^−/−^* double mutants, where adjacent internodes fail to separate from each other (11). Recent work has shown that microglia, the resident immune cells in the nervous system, phagocytose myelin abnormalities during development and into adulthood, thereby contributing to the establishment and maintenance of myelin integrity (4, 13, 14). However, the oligodendrocyte-intrinsic mechanisms that limit myelin abnormalities, especially during rapid axon ensheathment, remain incompletely understood.

The transcription factor EB (TFEB) is a basic helix–loop–helix (bHLH) leucine zipper transcription factor that is highly expressed in oligodendrocytes (15). Recent work has shown that TFEB strongly represses CNS myelination, with evolutionarily conserved roles across species (15–17). In the mammalian brain, TFEB acts as a molecular brake on CNS myelination by (1) inducing premyelinating oligodendrocyte apoptosis in a PUMA-BAX/BAK-dependent manner, and (2) inhibiting myelin sheath growth by repressing cholesterol biosynthesis, independent of its proapoptotic function (15, 18). Whether TFEB plays additional roles in myelin development, however, remains unclear.

Here, we identify an oligodendrocyte-intrinsic mechanism, orchestrated by the TFEB–autophagy axis, that limits myelin membrane protrusions and whorls at the onset of axon ensheathment, thereby regulating myelin integrity during developmental myelination. Using serial block-face scanning electron microscopy (SBEM) and a knock-in mouse line (*Enpp6-IRES-CreER^T2^*) that targets newly formed oligodendrocytes, we reconstructed early postnatal optic nerve myelination. We observe that newly formed myelin sheaths exhibit membrane protrusions and occasional degenerative myelin “whorls.” Conditional deletion of TFEB from newly formed oligodendrocytes significantly increases the density of these myelin membrane protrusions and whorls in the developing optic nerve. Mechanistically, TFEB directly binds to a cohort of autophagy genes and promotes autophagic flux in newly formed oligodendrocytes, thereby limiting myelin membrane protrusions and whorls independent of its previously described repressive role in myelination. Together, our findings identify a role for the TFEB–autophagy axis in establishing proper myelin during development, providing insights into the oligodendrocyte-intrinsic mechanisms that regulate myelin integrity.

## Results

### TFEB Is Expressed by Newly Formed Oligodendrocytes and Limits Myelin Whorl Number During Optic Nerve Development.

**Fig. 1.**
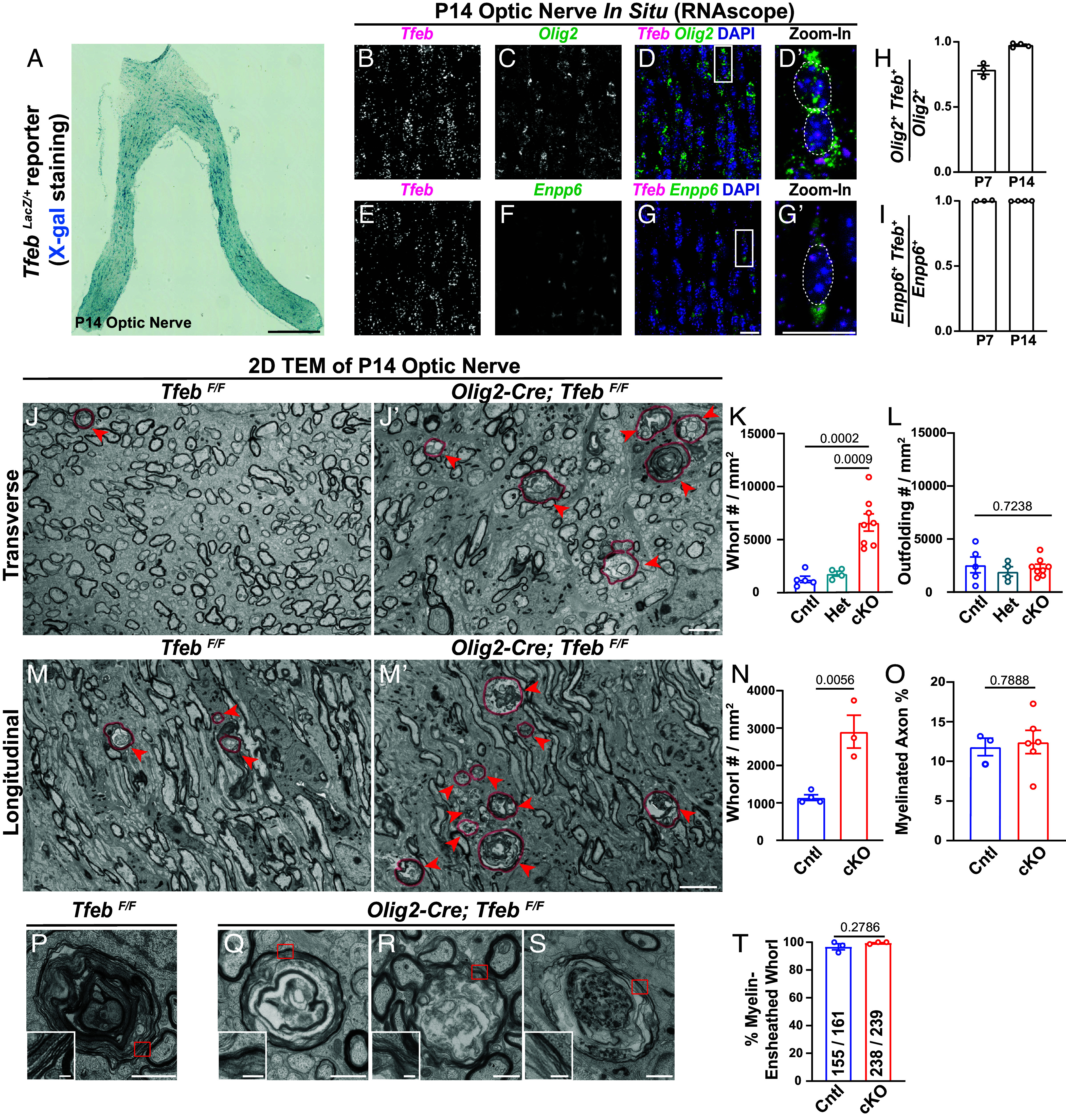
TFEB is expressed by newly formed oligodendrocytes and restricts myelin whorl number during optic nerve development. (*A*) Representative image of a longitudinal section from P14 *Tfeb^LacZ/+^* optic nerves, stained with X-gal. *n* = 3 animals. (*B*–*G*′) Double fluorescent in situ hybridization of P14 optic nerves using probes against *Tfeb* (magenta)*, Olig2* (green, top row), and *Enpp6* (green, bottom row). (*D*′) and (*G*′) represents enlarged views of the inset in *D* and *G*, respectively. (*H* and *I*) Quantification of the proportion of *Olig2^+^ Tfeb^+^* cells among total *Olig2^+^* cells (*H*) and the proportion of *Enpp6^+^ Tfeb^+^* cells among total *Enpp6^+^* cells in P7 and P14 optic nerves (*I*). (*J* and *J*′) Representative micrographs of transverse transmission electron microscopy (TEM) of P14 *Tfeb^F/F^* (*J*) and *Olig2-Cre; Tfeb^F/F^* optic nerves (*J*′), showing increased myelin whorl presence in *Olig2-Cre; Tfeb^F/F^* optic nerves (compare red arrowheads in *J* and *J*′). Whorls are outlined in red. (*K* and *L*) Quantification of myelin whorl (*K*) and outfolding density (*L*) in P14 *Tfeb^F/F^* (Cntl), *Olig2-Cre; Tfeb^F/+^* (Het), and *Olig2-Cre; Tfeb^F/F^* (cKO) optic nerves using transverse TEM sections. See also *SI Appendix*, Fig. S1 for myelin abnormality categories (*SI Appendix*, Fig. S1 *J*–*N*). (*M*–*M*′) Representative longitudinal TEM micrographs from P14 *Tfeb^F/F^* (*M*) and *Olig2-Cre; Tfeb^F/F^* optic nerves (*M*′). Red arrowheads indicate myelin whorls (outlined in red). (*N*) Quantification of whorl density in P14 *Tfeb* cKO and littermate control optic nerves on longitudinal TEM sections. (*O*) Quantification of myelinated axon percentage in P14 control and *Tfeb* cKO optic nerves using transverse TEM sections. (*P*–*S*) Representative transverse TEM micrographs from P14 *Tfeb^F/F^* (*P*) and *Olig2-Cre; Tfeb^F/F^* (*Q*–*S*), highlighting myelin sheaths surrounding whorls. *Insets* on the bottom left of (*P*–*S*) represent enlarged views of red boxes in *P*–*S*, respectively, revealing myelin sheaths surrounding whorls. (*T*) Quantification of the percentage of myelin-ensheathed whorls among total whorls in P14 control and *Tfeb* cKO optic nerves using transverse TEM sections. The number on each bar indicates the number of subsets of myelin-ensheathed whorls relative to the total number of whorls quantified. Error bars indicate SEM. Open circles in *H*, *I*, *K*, *L*, *N*, *O*, and *T* represent individual animals. One-way ANOVA followed by Tukey’s multiple comparisons test for *K* and *L*. Two-tailed Student’s *t* test for *N*, *O*, and *T*. (Scale bar, 500 μm in *A*; 10 μm in *G* for *B*–*G*; 10 μm in *G*′ for *D*′ and *G*′; 4 μm in *J*′ for *J* and *J*′; 5 μm in *M*′ for *M* and *M*′; 1 μm for *P*–*S*; 0.1 μm in the *Insets* of *P*; and 0.2 μm in the *Insets* of *Q*–*S*.)

To identify oligodendrocyte-intrinsic mechanisms governing myelin integrity, we leveraged TFEB, a premyelinating oligodendrocyte (pre-OL)-enriched transcription factor that has been implicated in myelin ensheathment (15, 16, 18). We first examined TFEB’s expression in early postnatal optic nerves at postnatal day 7 [P7; the beginning of retinal ganglion cell (RGC) axon ensheathment] and P14 (the peak of optic nerve myelination) (19). Using the *Tfeb^LacZ/+^* mouse line (15), we found numerous β-gal^+^ cells throughout the optic nerve at P14 (Fig. 1*A*). Double fluorescent in situ hybridization confirmed that *Tfeb* was highly expressed by oligodendrocyte lineage cells in the optic nerve: Approximately 79% of *Tfeb^+^* cells coexpressed *Olig2* at P7 (*SI Appendix*, Fig. S1 *A*–*C*′; quantified in Fig. 1*H*) and 97% at P14 (Fig. 1 *B*–*D*′; quantified in Fig. 1*H*). Moreover, *Tfeb* was expressed in nearly all newly formed oligodendrocytes, marked by *Enpp6* expression, at both P7 and P14 (Fig. 1 *E*–*G*′; quantified in Fig. 1*I* and *SI Appendix*, Fig. S1 *D*–*F*′).

To determine the role of TFEB in myelin ensheathment, we characterized *Olig2-Cre; Tfeb^F/F^* mutant optic nerves (hereafter referred to as *Tfeb* cKO), where TFEB is genetically deleted from oligodendrocyte lineage cells, using TEM. As expected, wild-type optic nerves displayed a range of abnormalities at P14, including myelin outfoldings, myelin whorls, and degenerative axons (*SI Appendix*, Fig. S1 *J*–*N*). Notably, *Tfeb* cKO optic nerves exhibited three-to-four-fold increases in myelin whorl density compared to littermate controls on both transverse TEM sections (Fig. 1 *J* and *J*′; quantified in Fig. 1*K*; 1,259.98 ± 301.02 whorls/mm^2^ in *Tfeb^F/F^*, 1,797.00 ± 239.54 whorls/mm^2^ in *Olig2-Cre; Tfeb^F/+^*, and 6,622.12 ± 810.96 whorls/mm^2^ in *Olig2-Cre; Tfeb^F/F^* optic nerves) and longitudinal TEM sections (Fig. 1 *M* and *M*′; quantified in Fig. 1*N*), alongside significantly increased myelin sheath thickness (*SI Appendix*, Fig. S1 *G*–*I*). The myelin whorl phenotype was not accompanied by any changes in the density of myelin outfoldings (Fig. 1*L*) or the percentage of normally myelinated axons (Fig. 1*O*), suggesting a distinct mechanism underlying the formation and elimination of myelin whorls.

### The Majority of Myelin Whorls Are Located Within the Cytoplasm of Oligodendrocytes and Are Associated with Myelinated Axons.

**Fig. 2.**
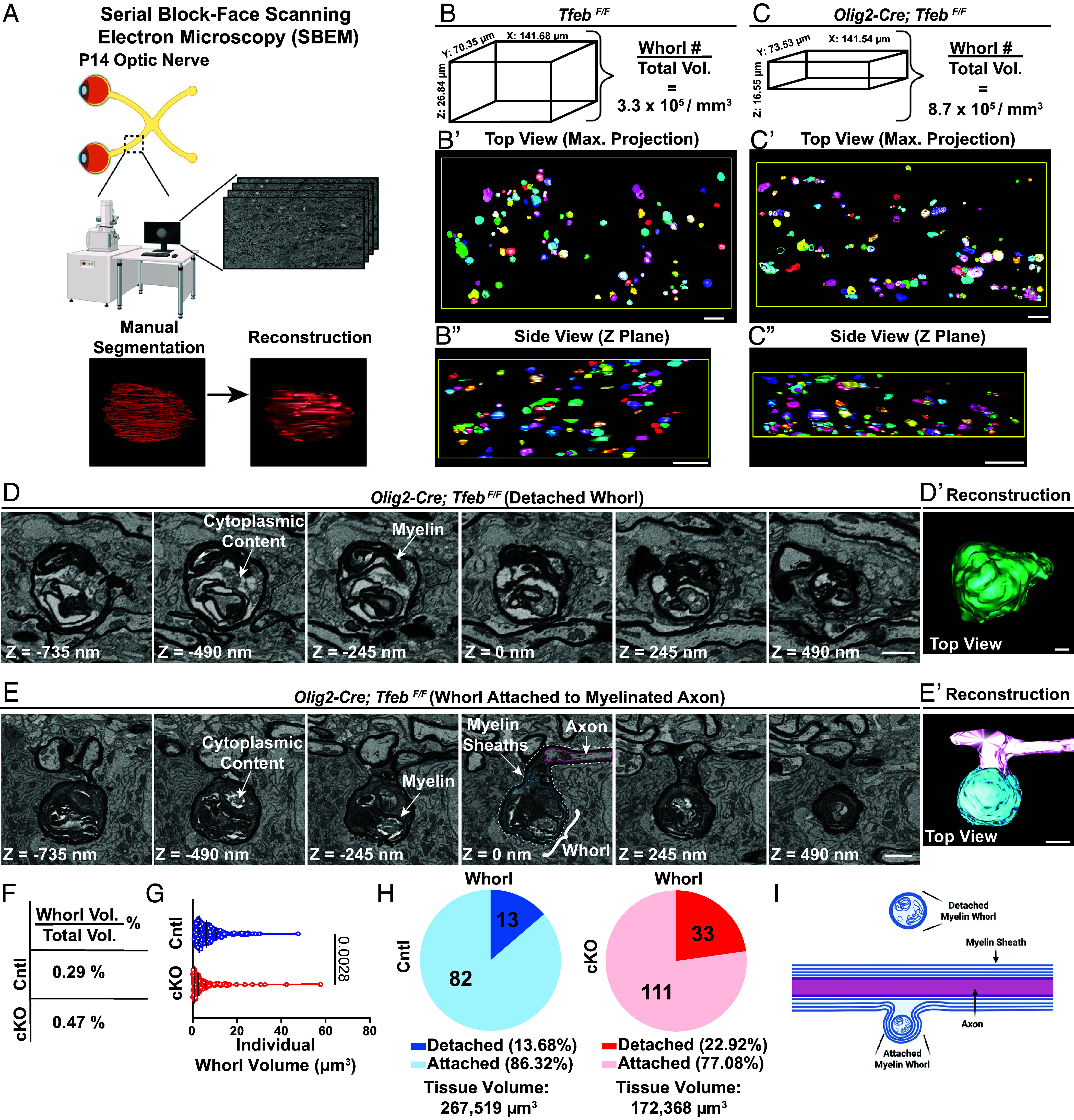
The majority of myelin whorls are located within oligodendrocyte cytosol and are associated with myelinated axons, as revealed by SBEM. (*A*) Workflow for SBEM imaging and analysis of P14 optic nerves, including tissue preparation, SBEM imaging (Z-step size: 35 nm), manual segmentation, and 3D reconstruction. Created in BioRender. L. Sun (2026) https://BioRender.com/id3onr3. (*B*–*C*″) SBEM reconstruction of P14 *Tfeb^F/F^* (*B*–*B*″) and *Olig2-Cre; Tfeb^F/F^* optic nerves (*C*–*C*″). (*B*) and (*C*) show the SBEM volume sizes and whorl densities from *Tfeb^F/F^* and *Olig2-Cre; Tfeb^F/F^* optic nerves, respectively. The reconstructed myelin whorls can be visualized by maximum projections from the top view (*B*′ and *C*′) and side view (*B*″ and *C*″) in *Tfeb^F/F^* (*B*′ and *B*″) and *Olig2-Cre; Tfeb^F/F^* optic nerves (*C*′ and *C*″). (Movies S1–S4). (*D* and *D*′) Representative SBEM sections along the z-axis of a detached whorl (*D*), and its reconstruction (*D*′) in the *Olig2-Cre; Tfeb^F/F^* optic nerve. (*E* and *E*′) Representative SBEM sections along the z-axis of a whorl associated with a myelinated axon (*E*), and its reconstruction (*E*′) in the *Olig2-Cre; Tfeb^F/F^* optic nerve. The myelin whorl is outlined in blue, while the myelinated axon is outlined in magenta. The dashed line outlines the myelin sheaths that surround the axon and myelin whorl. *SI Appendix*, Fig. S2*A* and Movies S5–S8. (*F*) Quantification of the proportion of total whorl volume to the total optic nerve volume imaged by SBEM in P14 *Tfeb^F/F^* (Cntl) and *Olig2-Cre; Tfeb^F/F^* (cKO) optic nerves. (*G*) Quantification of individual whorl size in P14 control and *Tfeb* cKO optic nerves. Black lines indicate the median, and dashed lines represent the quartile values of individual whorl volumes. (*H*) Quantification of “detached” (13.68% in control, 22.92% in *Tfeb* cKO) and “attached” whorl percentage (86.32% in control, 77.08% in *Tfeb* cKO). The numbers on the pie charts represent the reconstructed myelin whorl numbers under each category. (*I*) Schematics showing detached myelin whorls and myelin whorls associated with myelinated axons. Created in BioRender. L. Sun (2026) https://BioRender.com/id3onr3. See more examples in *SI Appendix*, Fig. S2*A*. Open circles in (*G*) represent individual whorls. Unpaired Student’s *t* test for *G*. (Scale bar, 10 μm for *B*′, *B*″, *C*′ and *C*″; and 1 μm in *D*, *D*′, *E*, and *E*′).

Degenerative myelin whorls are frequently found during demyelination (7), but what is the identity of development-associated myelin whorls? Ultra-high-resolution TEM images revealed that nearly all myelin whorls in both *Tfeb* control and cKO were enwrapped by intact myelin sheaths (Fig. 1 *P–S*; quantified in Fig. 1*T*), suggesting that myelin whorls are associated with normally formed myelin sheaths. To reveal the full geometry and content of myelin whorls in volume, we conducted SBEM on P14 control and *Tfeb* cKO optic nerves. After manual segmentation and 3D reconstruction (Fig. 2*A*), we identified 95 whorls within a volume of 267,519 μm^3^ in a control optic nerve (Fig. 2 *B*–*B*″ and Movies S1 and S2). In sharp contrast, the *Tfeb* cKO optic nerve exhibited a 2.6-fold increase in myelin whorl density compared to the control (Fig. 2 *C*–*C*″; 144 whorls in a volume of 172,368 μm^3^; Movies S3 and S4). At a higher magnification, we found that whorls contained cytoplasmic and myelin contents in both control and *Tfeb* cKO optic nerves (*SI Appendix*, Fig. S2*A* for *Tfeb* control; Fig. 2 *D* and *E* for *Tfeb* cKO). The fraction of whorl volume relative to total tissue volume, reconstructed by SBEM, also increased in the *Tfeb* cKO optic nerve compared to the control (Fig. 2*F*). Meanwhile, we observed numerous whorl clusters in the *Tfeb* cKO optic nerve (Fig. 2 *C′* and *C″*), with individual whorls significantly smaller than those in the control (Fig. 2*G*).

To determine the spatial relationship between whorls and myelinated axons, we analyzed 767 SBEM sections from the control optic nerve and 473 SBEM sections from the *Tfeb* cKO optic nerve. We identified two distinct types of whorls based on their association with myelinated axons. A small subset of whorls was dissociated from myelinated axons (Fig. 2*D* and *Top* panel in *SI Appendix*, Fig. S2*A*; quantified in Fig. 2*H*), appearing as “ball-like” structures revealed by the 3D reconstruction (Fig. 2*D*′ and *Top* panel in *SI Appendix*, Fig. S2*A* and Movies S5 and S7). Surprisingly, 86.32% of whorls in control and 77.08% of whorls in the *Tfeb* cKO optic nerve were attached to myelinated axons (Fig. 2 *E* and *E*′ and *Bottom* panel in *SI Appendix*, Fig. S2*A*; quantified in Fig. 2*H* and Movies S6 and S8).

To further examine the subcellular localization of whorls associated with myelinated axons, we analyzed high-resolution TEM images. Indeed, these whorls were surrounded by compact myelin sheaths and were associated with axons (Fig. 1 *P*–*T* and *SI Appendix*, Fig. S2 *B* and *C*). Moreover, whorls were found between the oligodendrocyte plasma membrane and the compact myelin sheaths, indicating that they are located within an oligodendrocyte, but not within the axon plasma membrane or periaxonal extracellular space (*SI Appendix*, Fig. S2 *B*–*D*; see schematic in Fig. 2*I*). Thus, *Tfeb* cKO optic nerves exhibit smaller myelin whorls in volume but a greater number overall, the majority of which are associated with myelinated axons.

### TFEB Functions Cell-Autonomously in Newly Formed Oligodendrocytes to Remodel Myelin Membrane and Limit Whorl Number During the Initiation of Axon Ensheathment.

In the murine brain, TFEB functions as a molecular brake on myelination by inducing premyelinating oligodendrocyte apoptosis and separately inhibiting myelin sheath thickness (15). To investigate whether the increased whorl density in *Tfeb* cKO optic nerve is due to aberrantly increased oligodendrocyte cell number, we immunostained P14 optic nerves using the CC1 antibody (a postmitotic oligodendrocyte marker). In contrast to previous observations in the brain (15), *Tfeb* cKO optic nerves showed no change in CC1^+^ cell density compared to littermate controls (*SI Appendix*, Fig. S3 *A* and *A*′; quantified in Fig. S3*B*). Moreover, Olig2^+^ (pan-oligodendrocyte lineage marker) and PDGFRα^+^ [oligodendrocyte precursor cell (OPC) marker] cell densities remained unchanged in *Tfeb* cKO optic nerves (*SI Appendix*, Fig. S3 *C*–*F*), together showing that optic nerve oligodendrogenesis is not affected in the absence of TFEB.

To determine whether aberrantly enhancing oligodendrocyte numbers can increase the density of whorls, we analyzed the optic nerves from *Olig2-Cre; Bax^F/F^; Bak^−/−^* mutants in which oligodendrocyte apoptosis is genetically abolished (15). Indeed, *Olig2-Cre; Bax^F/F^; Bak^−/−^* optic nerves exhibited a significant increase in CC1^+^ cell density compared to controls (*Bax^F/F^; Bak^−/−^*), demonstrating that oligodendrocyte apoptosis is successfully perturbed in the optic nerve using this strategy (*SI Appendix*, Fig. S3 *G* and *G*′; quantified in *SI Appendix*, Fig. S3*H*). However, *Olig2-Cre; Bax^F/F^; Bak^−/−^* mutants exhibited unchanged whorl densities (*SI Appendix*, Fig. S3 *I* and *I*′; quantified in *SI Appendix*, Fig. S3*J*) and normal myelin sheath thickness compared to controls at P14 (*SI Appendix*, Fig. S3 *K* and *L*).

**Fig. 3.**
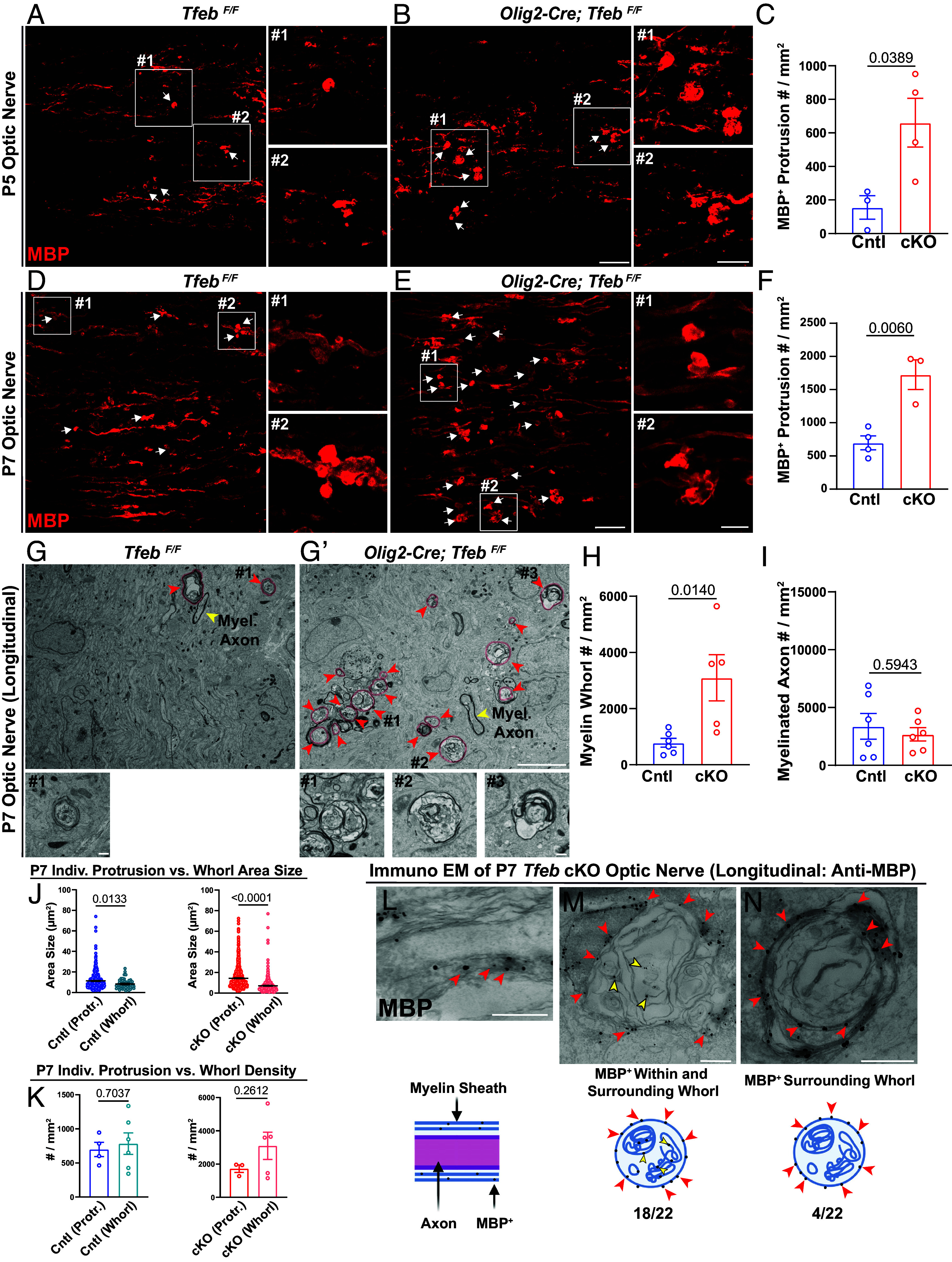
TFEB limits the number of myelin protrusions and whorls at the onset of optic nerve myelination. (*A* and *B*) Representative micrographs of P5 *Tfeb^F/F^* (*A*) and *Olig2-Cre; Tfeb^F/F^* (*B*) optic nerve sections stained with an antibody raised against MBP. White arrows indicate MBP^+^ protrusions extended from longitudinally formed processes. White bounding boxes labeled #1 and #2 indicate the enlarged views of the insets shown in *A* and *B*. (*C*) Quantification of MBP^+^ protrusion density in P5 *Tfeb^F/F^* (Cntl) and *Olig2-Cre; Tfeb^F/F^* (cKO) optic nerves (30-μm thickness). (*D* and *E*) Representative micrographs of P7 *Tfeb^F/F^* (*D*) and *Olig2-Cre; Tfeb^F/F^* (*E*) optic nerve sections stained by an MBP antibody. White arrows indicate MBP^+^ protrusions. White bounding boxes labeled #1 (single MBP^+^ protrusions) and #2 (rosette-like MBP^+^ formations) indicate the enlarged views of the insets shown in *D* and *E*. (*SI Appendix*, Fig. S4 *A*–*G* and Movies S9–S11). (*F*) Quantification of MBP^+^ protrusion density in P7 *Tfeb^F/F^* (Cntl) and *Olig2-Cre; Tfeb^F/F^* (cKO) optic nerves (20-μm thickness). (*G* and *G*′) Representative longitudinal TEM micrographs of P7 *Tfeb^F/F^* (*G*) and *Olig2-Cre; Tfeb^F/F^* (*G*′) optic nerves. Red arrowheads indicate myelin whorls (outlined in red). Yellow arrowheads indicate myelinated axons. (*H* and *I*) Quantification of myelin whorl density (*H*) and myelinated axon density (*I*) in P7 *Tfeb^F/F^* (Cntl) and *Olig2-Cre; Tfeb^F/F^* (cKO) optic nerves. (*J*) Quantification and comparison of individual MBP^+^ protrusion area by immunostaining and individual myelin whorl area by TEM in P7 *Tfeb^F/F^* (*Left*) and *Olig2-Cre; Tfeb^F/F^* (*Right*) optic nerves. (*K*) Quantification and comparison of protrusion density and myelin whorl density in P7 *Tfeb^F/F^* (*Left*) and *Olig2-Cre; Tfeb^F/F^* (*Right*) optic nerves. (*L*–*N*) Immuno-EM of MBP labeling in P7 *Olig2-Cre; Tfeb^F/F^* optic nerves shows electron-dense silver-enhanced DAB deposits marking MBP^+^ myelin. Red arrowheads in *L*, *M*, and *N* indicate MBP^+^ particles. Yellow arrowheads in *M* indicate MBP^+^ particles within a whorl. Created in BioRender. L. Sun (2026) https://BioRender.com/id3onr3. Error bars indicate SEM. Open circles in *C*, *F*, *H*, *I*, and *K* represent individual animals. Open circles in *J* represent individual MBP^+^ protrusions or whorls. Two-tailed Student’s *t* test for *C*, *F*, *H*–*K*. (Scale bar, 20 μm in *B* for *A* and *B*; 20 μm in *E* for *D* and *E*; 5 μm in *Inset* #2 of *B* for *Insets* pertaining to *A* and *B*; 5 μm in *Inset* #2 of *E* for *Insets* pertaining to *D* and *E*; 4 μm in *G*′ for *G* and *G*′; 1 μm in *Inset* #1 pertaining to *G*; 1 μm in *Insets* #3 for *Insets* #1, #2, and #3 pertaining to *G*′; and 500 nm in *L*–*N*.)

Given that axon ensheathment is established rapidly in the developing optic nerve and requires large amounts of myelin (19), we hypothesized that myelin whorls arise during early axon ensheathment. We labeled oligodendrocytes and their newly formed myelin sheaths using a myelin basic protein (MBP) antibody at P5, P7, and P14, on and before the whorl phenotype observed by TEM at P14. MBP immunolabeling revealed rapid oligodendrocyte differentiation and axon ensheathment in early postnatal optic nerves. At P5, very few MBP^+^ differentiating oligodendrocytes were observed (*Top* panel in *SI Appendix*, Fig. S4*A*). MBP^+^ oligodendrocytes and their processes were prominent at P7 and rapidly covered the entire optic nerve by P14 (*Middle* and *Bottom* panels in *SI Appendix*, Fig. S4*A*). Notably, between P5 and P7, when oligodendrocytes begin to ensheathe RGC axons, numerous MBP^+^ protrusions emerged from longitudinally formed MBP^+^ processes in control optic nerves (Fig. 3 *A* and *D*; quantified in Fig. 3 *C* and *F* and *SI Appendix*, Fig. S4 *C* and *D*). These MBP^+^ protrusions encompassed both single MBP^+^ protrusions and rosette-like structures extending from MBP^+^ processes (*Insets* in Fig. 3*D* and Movies S9–S11). In contrast, *Tfeb* cKO optic nerves exhibited a 2- to 4-fold increase in MBP^+^ protrusion density at P5 and P7 (Fig. 3 *B* and *E*; quantified in Fig. 3 *C* and *F*; *SI Appendix*, Fig. S4 *C′ and D′*). Importantly, these MBP^+^ protrusions were not cryosectioning artifacts or oligodendrocyte somata, as evidenced by multichannel confocal imaging in orthogonal views and three-dimensional renderings (*SI Appendix*, Fig. S4 *F* and *G* and Movies S9–S11).

The increased density of MBP^+^ protrusions in P5 and P7 *Tfeb* cKO optic nerves suggests that myelin whorls are readily present during early axonal ensheathment. To test this, we conducted TEM imaging on P7 *Tfeb* control and cKO optic nerves on the longitudinal plane. At P7, very few axons were myelinated, and minimal but detectable myelin whorls were observed in control optic nerves (Fig. 3*G*; quantified in Fig. 3*H*). In contrast, *Tfeb* cKO optic nerves exhibited a significantly increased number of myelin whorls (Fig. 3*G*′; quantified in Fig. 3*H*; 782.68 ± 157.55 whorl/mm^2^ in control optic nerves, 3097.62 ± 821.02 whorls/mm^2^ in *Tfeb* cKO optic nerves), but a similar density of myelinated axons compared to controls (Fig. 3*I*). Like at P14, we also observed myelin whorls that were directly associated with myelinated axons in P7 *Olig2-Cre; Tfeb^F/F^* optic nerves (*SI Appendix*, Fig. S4*H*). Intriguingly, the densities of myelin whorls were comparable to those of MBP^+^ protrusions (Fig. 3*K*), while individual myelin whorl areas were significantly smaller than MBP^+^ protrusion areas (Fig. 3*J*). Using immuno-EM labeling for MBP, we found that all myelin whorls (22 of 22) in the P7 *Olig2-Cre; Tfeb^F/F^* optic nerve were encapsulated by MBP^+^ myelin sheaths, the majority of which (18 of 22) contained electron-dense, silver-gold enhanced DAB/GSSP deposits that marked MBP^+^ myelin particles within the whorls (Fig. 3 *L*–*N*). These results suggest that the MBP^+^ myelin membrane protrusions and whorls are correlated.

**Fig. 4.**
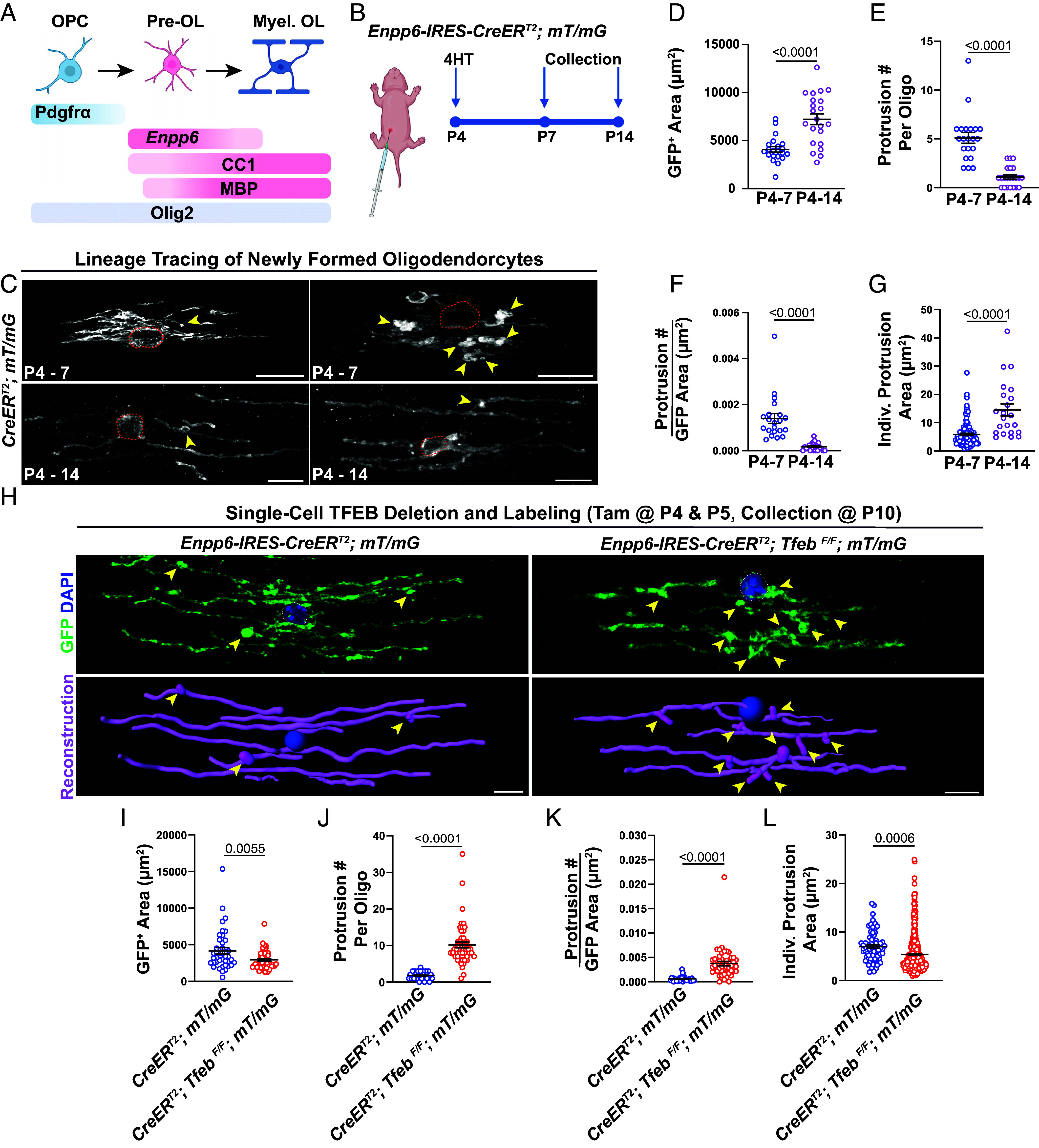
TFEB functions cell-autonomously in newly formed oligodendrocytes to limit myelin protrusions in the developing optic nerve. (*A*) Diagram demonstrating oligodendrocyte stepwise differentiation and the markers for OPC, premyelinating oligodendrocyte (Pre-OL), and myelinating oligodendrocyte (Myel. OL). Created in BioRender. L. Sun (2026) https://BioRender.com/id3onr3. (*B*) Schematics using the *Enpp6-IRES-CreER^T2^; mT/mG* mouse line to demarcate newly formed oligodendrocytes and their myelin sheaths with membrane-tethered GFP. (*C*) Representative micrographs of lineage-traced oligodendrocytes in *Enpp6-IRES-CreER^T2^; mT/mG* optic nerves at P7 (*Top*) and P14 (*Bottom*), following 4HT injections at P4. Red dashed lines outline oligodendrocyte cell bodies, and the yellow arrows indicate GFP^+^ protrusions (16-μm thickness). (*D*–*G*) Quantifications of GFP^+^ area per oligodendrocyte (*D*), protrusion number per labeled oligodendrocyte (*E*), normalization of protrusion number to the total GFP^+^ area (*F*), and individual protrusion areas (*G*). (*H*) Representative fluorescence images (green) and corresponding Imaris 3D reconstructions (magenta) of *Enpp6-IRES-CreER^T2^; mT/mG* (*Left*) and *Enpp6-IRES-CreER^T2^; Tfeb^F/F^; mT/mG* oligodendrocytes (*Right*). White dashed lines outline oligodendrocyte cell bodies, and the yellow arrowheads indicate GFP^+^ protrusions associated with myelin sheaths. (*I*–*L*) Quantification of GFP^+^ area per oligodendrocyte (*I*), protrusion number per labeled oligodendrocyte (*J*), normalization of protrusion number to the total GFP^+^ area (*K*), and individual protrusion areas (*L*). Error bars indicate SEM. Open circles in *D*–*F* and *I*–*K* represent individual cells from three animals per genotype. Open circles in *G* and *L* represent individual protrusions. Two-tailed Student’s *t* test for *D*–*G* and *I*–*L*. (Scale bar, 20 μm for *C* and *H*.)

To determine whether TFEB is cell-autonomously required by newly formed oligodendrocytes to limit myelin membrane protrusions, we employed a mouse line (*Enpp6-IRES-CreER^T2^*) that specifically labels pre-OLs and subsequently differentiated newly formed oligodendrocytes (Fig. 4*A*) (20). We crossed *Enpp6-IRES-CreER^T2^* mice with the *mT/mG* reporter line to sparsely label newly formed oligodendrocytes and their myelin sheaths with membrane-tethered GFP following 4-hydroxytamoxifen (4HT) injections at P4 (Fig. 4*B*). 3 d postinjection (P7), genetically labeled oligodendrocytes began elaborating longitudinally formed processes, along with numerous GFP^+^ protrusions associated with the myelin sheaths (top row in Fig. 4*C*; quantified in Fig. 4 *D*–*G*). 10 d after 4HT injection (P14), myelin sheaths were further elongated, and the density of GFP^+^ protrusions was significantly reduced but individual protrusion sizes were increased compared to P7 (bottom row in Fig. 4*C*; quantified in Fig. 4 *D*–*G*), suggesting a myelin membrane remodeling process during axon ensheathment.

Next, we genetically deleted TFEB from newly formed oligodendrocytes while concurrently labeling them, using the *Enpp6-IRES-CreER^T2^* driver line. As expected, control oligodendrocytes (*Enpp6-IRES-CreER^T2^; mT/mG*) that were lineage-traced from P4 exhibited few GFP^+^ protrusions by P10 (*Left* panel in Fig. 4*H*). In contrast, *Enpp6-IRES-CreER^T2^; Tfeb^F/F^; mT/mG* mutant oligodendrocytes displayed smaller membrane areas but significantly increased numbers of GFP^+^ protrusions compared with control cells (*Right* panel in Fig. 4*H*; quantified in Fig. 4 *I*–*K*). Interestingly, individual protrusions were smaller in *Enpp6-IRES-CreER^T2^; Tfeb^F/F^; mT/mG* mutant oligodendrocytes compared with those in control cells (Fig. 4*L*). Thus, TFEB expressed by newly formed oligodendrocytes reduces the number of myelin membrane protrusions and myelin whorls during the onset of axon ensheathment.

### TFEB Enhances Autophagic Flux to Reduce Myelin Membrane Protrusions and Myelin Whorls.

**Fig. 5.**
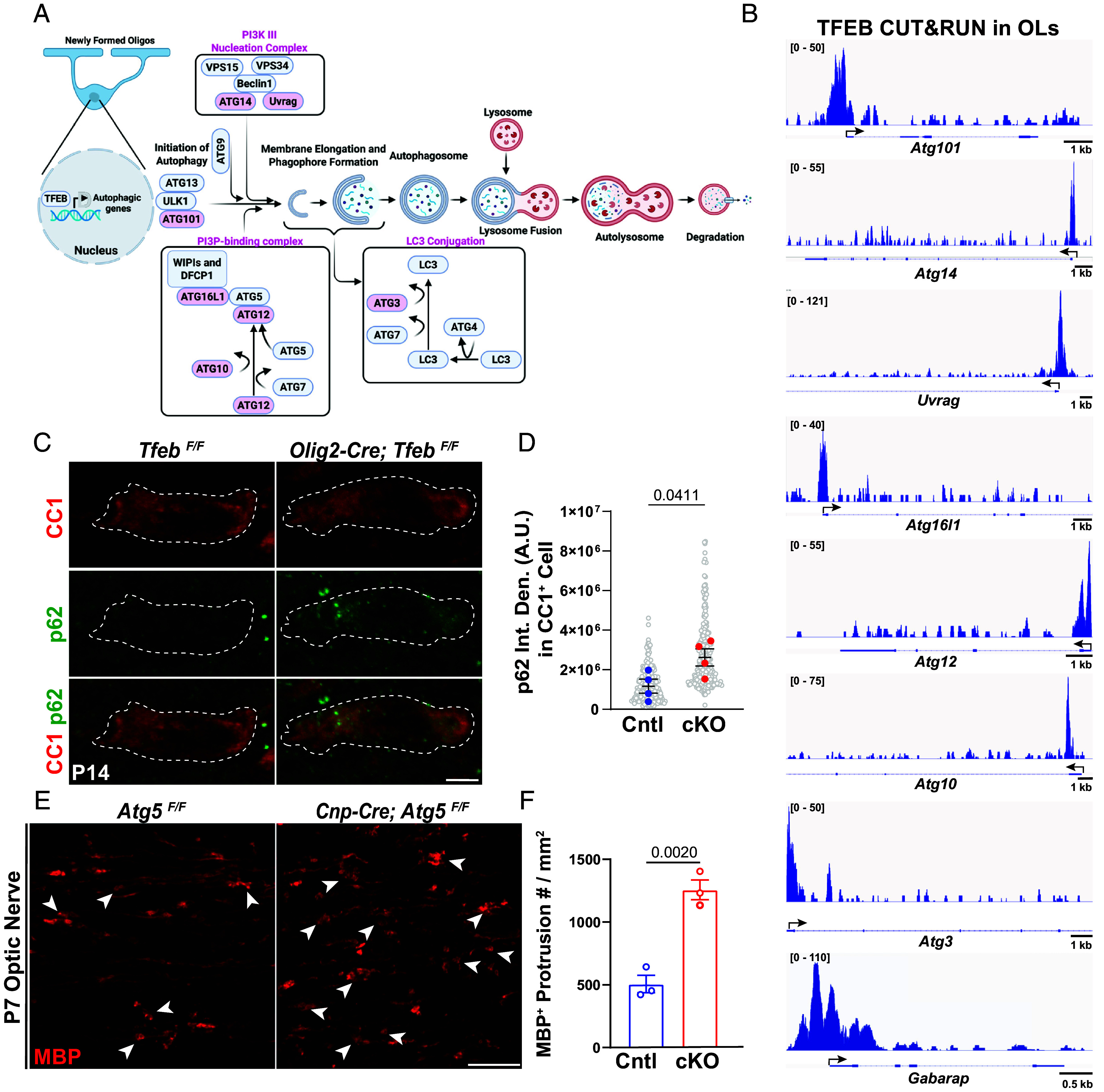
TFEB activates autophagic flux to reduce MBP^+^ protrusions in newly formed oligodendrocytes during axon ensheathment. (*A*) Diagram showing the autophagy pathway in newly formed oligodendrocytes, including autophagy initiation, autophagosome elongation, and fusion with the lysosome. Schematic created in BioRender. L. Sun (2026) https://BioRender.com/id3onr3 and modified from Hansen et al., 2018 (23). (*B*) Genome browser view of TFEB binding sites in autophagy genes, highlighted in red (*A*). Black arrows indicate transcription start sites. (*C*) Double immunofluorescence staining against CC1 and p62 in *Tfeb^F/F^* (*Left*) and *Olig2-Cre; Tfeb^F/F^* (*Right*) optic nerves at P14. Dashed lines demarcate CC1^+^ oligodendrocyte cell bodies. (*D*) Quantification of integrated intensity (arbitrary units, A.U.) of p62 immunofluorescence signals in CC1^+^ cells in P14 *Tfeb^F/F^* (Cntl) and *Olig2-Cre; Tfeb^F/F^* (cKO) optic nerves. Blue and red circles indicate individual animals. *n* = 4 animals. Gray circles indicate individual CC1^+^ cells used for quantification of p62 immunofluorescence signals. (*E*) Representative images of P7 *Atg5^F/F^* and *Cnp-Cre; Atg5^F/F^* optic nerve sections stained with an MBP antibody. White arrowheads indicate MBP^+^ protrusions. (*F*) Quantification of MBP^+^ protrusion density in P7 *Atg5^F/F^* (Cntl) and *Cnp-Cre; Atg5^F/F^* (cKO) (20-μm thickness). Error bars indicate SEM. Open circles in *F* represent individual animals. Two-tailed Student’s *t* test for *D* and *F*. (Scale bar, 5 μm in *C* and 20 μm in *E*.)

What are the molecular mechanisms by which TFEB limits the number of myelin membrane protrusions and whorls? TFEB is known for its roles in promoting autophagy in a variety of cell types following starvation and stressed conditions, thereby clearing unwanted cellular contents and organelles (Fig. 5*A*) (21, 22). To test whether TFEB activates autophagy in newly formed oligodendrocytes during axon ensheathment, we first investigated the direct binding targets of TFEB in oligodendrocytes. We mined our TFEB CUT and RUN dataset of oligodendrocytes and found that TFEB directly binds to a cohort of autophagy genes involved in every step of autophagy flux (Fig. 5*B*) (18). These genes include *Atg101* (autophagy initiation), *Atg14* and *Uvrag* (PI3K III nucleation complex), *Atg16l1*, *Atg12*, and *Atg10* (PI3P-binding complex), and *Atg3* and *Gabarap* (LC3 conjugation; see highlights in Fig. 5*A*). Next, we characterized *Tfeb* cKO optic nerves using SQSTM1/p62 (a known substrate of autophagy) and CC1 antibodies at P14. CC1^+^ oligodendrocytes in *Tfeb* cKO optic nerves exhibited a significant increase in p62 immunoreactivity compared to controls (Fig. 5*C*; quantified in Fig. 5*D*), indicating that autophagy flux is indeed impaired in *Tfeb* cKO oligodendrocytes.

**Fig. 6.**
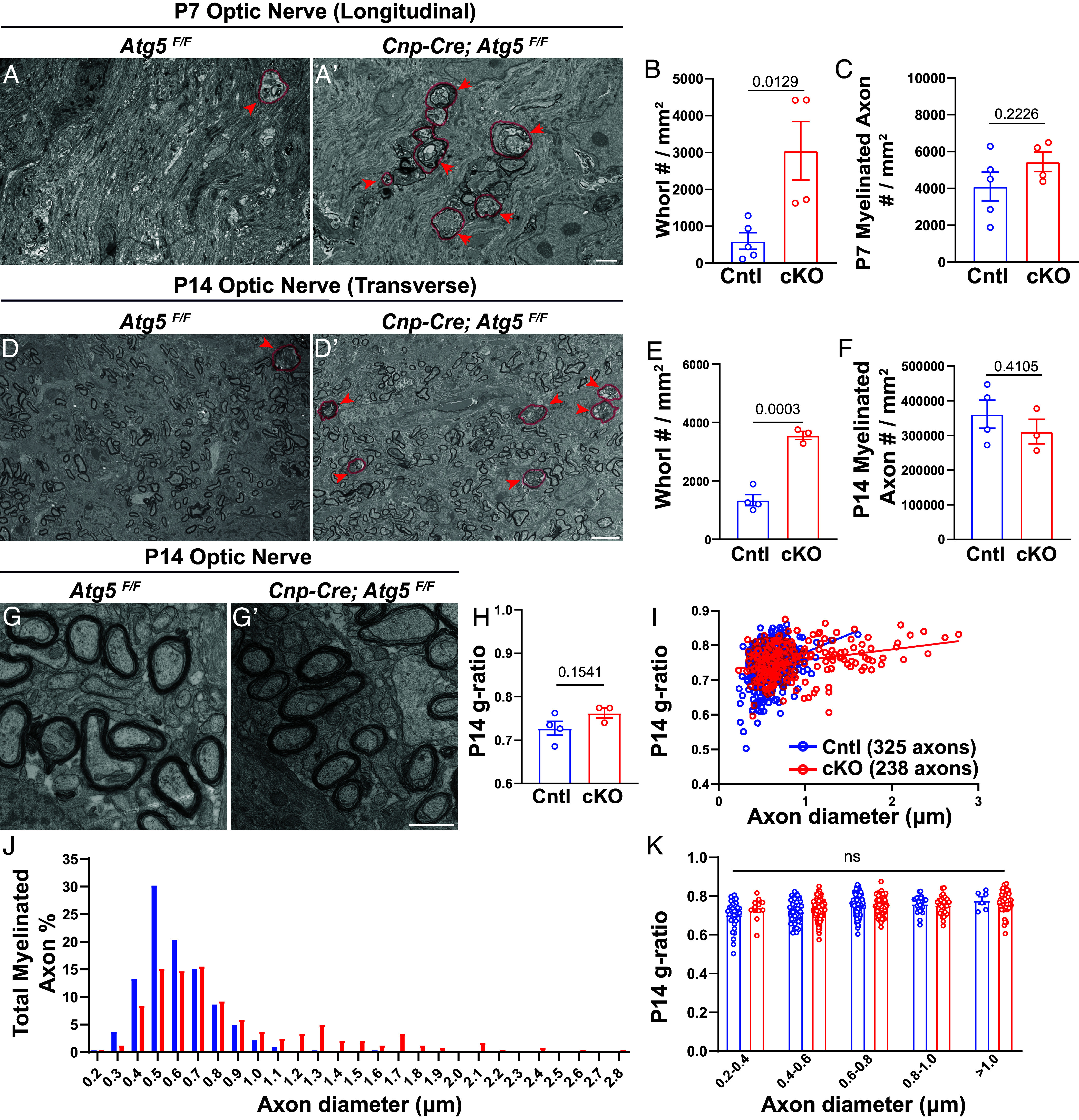
Genetic blockade of autophagy in oligodendrocytes increases myelin whorl density but does not affect myelin sheath thickness during optic nerve development. (*A* and *A*′) Representative longitudinal TEM micrographs from *Atg5^F/F^* (*A*) and *Cnp-Cre; Atg5^F/F^* (*A*′) optic nerves at P7. Red arrowheads indicate whorls (outlined in red). (*B* and *C*) Quantification of whorl density (*B*) and myelinated axon density (*C*) in P7 *Atg5^F/F^* (Cntl) and *Cnp-Cre; Atg5^F/F^* (cKO) optic nerves, using longitudinal TEM sections. (D and D′) Representative transverse TEM micrographs from *Atg5^F/F^* (*D*) and *Cnp-Cre; Atg5^F/F^* (*D*′) optic nerves at P14. Red arrowheads indicate whorls (outlined in red). (*E* and *F*) Quantification of whorl density (*E*) and myelinated axon density (*F*) in P14 *Atg5^F/F^* (Cntl) and *Cnp-Cre; Atg5^F/F^* (cKO) optic nerves, using transverse TEM sections. (*G* and *G*′) Representative TEM micrographs from *Atg5^F/F^* (*G*) and *Cnp-Cre; Atg5^F/F^* (G′) optic nerves at P14. (*H* and *I*) Quantification of average *g-*ratio (*H*) and a scatter plot showing *g*-ratios of myelinated axons as a function of axon diameter (*I*) in P14 *Atg5^F/F^* (Cntl: Blue) and *Cnp-Cre; Atg5^F/F^* (cKO: Red) optic nerves. (*J* and *K*) Total percentages of myelinated axons with different diameters from all animals (*J*), and *g*-ratios categorized by axon diameter (*K*), for *Atg5^F/F^* (Cntl) and *Cnp-Cre; Atg5^F/F^* (cKO) optic nerves at P14. Error bars indicate SEM. Open circles in *B*, *C*, *E*, *F*, and *H* represent individual animals. Open circles in *I* and *K* represent individual axons. Two-tailed Student’s *t* test for *B*, *C*, *E*, *F*, *H*, and *K*. (Scale bar, 2 μm in *A*′ for *A* and *A*′; 4 μm in *D*′ for *D* and *D*′; and 1 μm in *G*′ for *G* and *G*′.)

To test whether autophagy is required for myelin membrane remodeling and myelin whorl reduction, we generated the *Cnp-Cre; Atg5^F/F^* conditional knockout mouse line in which *Atg5*, a critical gene required for autophagosome elongation, was deleted from pre-OLs and subsequently differentiated myelinating oligodendrocytes (hereafter referred to as *Atg5* cKO). As expected, autophagy flux was disrupted in *Atg5* cKO oligodendrocytes from P14 optic nerves, as indicated by significantly elevated p62 fluorescence levels compared to controls (*SI Appendix*, Fig. S5*A*; quantified in *SI Appendix*, Fig. S5*B*). At P7, when axon ensheathment has just begun (*SI Appendix*, Fig. S4), *Atg5* cKO exhibited a significant increase in the density of MBP^+^ protrusions compared to littermate controls (Fig. 5*E*; quantified in Fig. 5*F*). This phenotype was accompanied by a substantial increase in myelin whorl density, as revealed by TEM at both P7 (Fig. 6 *A* and *A*′; quantified in Fig. 6*B*) and P14 (Fig. 6 *D* and *D*′; quantified in Fig. 6*E*), whereas oligodendrocyte number and myelinated axon density remained unchanged in *Atg5* cKO optic nerves (Fig. 6 *C* and *F* and *SI Appendix*, Fig. S5 *E* and *F*). Importantly, *Tfeb* cKO and *Atg5* cKO mutants were indistinguishable in both MBP^+^ protrusion density and myelin whorl density at P7 (*SI Appendix*, Fig. S5 *C* and *D*), strongly suggesting that TFEB and autophagy act in the same pathway to restrict myelin membrane protrusions and whorls.

Our previous work shows that TFEB potently represses myelin sheath growth (15, 18). Indeed, *Tfeb* cKO optic nerves exhibited significantly increased myelin sheath thickness (*SI Appendix*, Fig. S1 *G*–*I*). To test whether the increased number of myelin membrane protrusions and whorls in *Tfeb* cKO optic nerves was due to enhanced myelination, we characterized myelin sheath thickness of *Atg5* cKO optic nerves. In contrast to the hypermyelinated *Tfeb* cKO optic nerves, *Atg5* cKO optic nerves exhibited no change in myelin sheath thickness compared to controls at P14, as measured by *g*-ratio (Fig. 6 *G* and *G*′, quantified in Fig. 6 *H* and *I*). We observed an increase in the proportion of large-diameter axons that were myelinated in *Atg5* cKO optic nerves (Fig. 6*J*). However, the *g*-ratio across multiple axon calibers remained unchanged in *Atg5* cKO optic nerves compared to controls (Fig. 6*K*). Thus, although TFEB is a potent repressor of myelin sheath growth, its role in autophagy-dependent myelin membrane remodeling and whorl reduction represents a separate function in newly formed oligodendrocytes.

### *Tfeb* and *Atg5* cKO Mice Exhibit Impaired Visual Function.

**Fig. 7.**
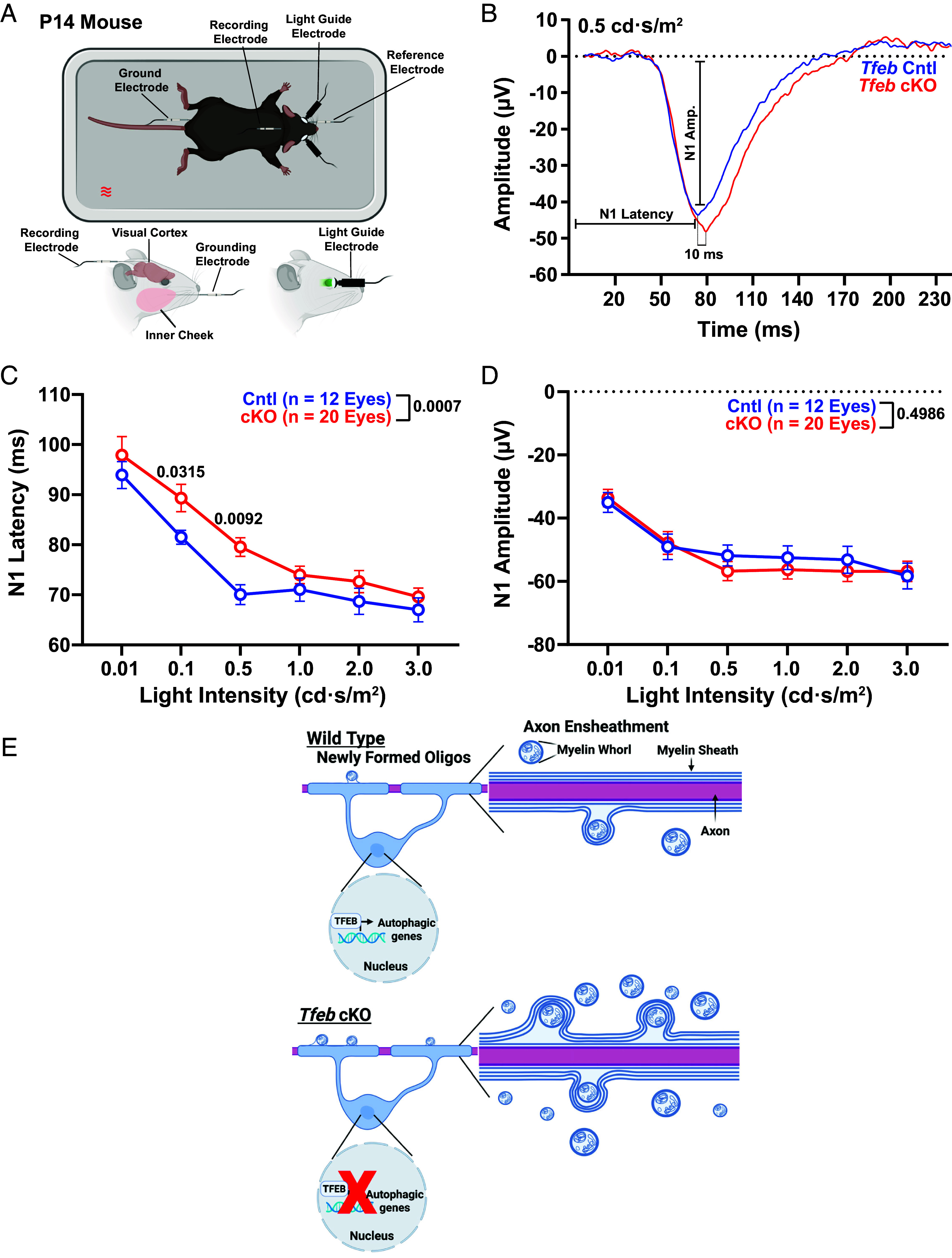
*Tfeb* cKO mice exhibit impaired visual function at the peak of myelin whorl presence. (*A*) Schematic of the VEP recording performed on P14 mice. Created in BioRender. L. Sun (2026) https://BioRender.com/id3onr3. (*B*) Representative VEP traces of control and *Tfeb* cKO under 0.5 cd s/m^2^ light intensity, showing a 10-millisecond delay of the N1 peak in *Tfeb* cKO mice compared to the control. (*C* and *D*) VEP N1 latencies (*C*) and amplitudes (*D*) across 0.01, 0.1, 0.5, 1.0, 2.0, and 3.0 cd s/m^2^ light intensities. *n* = 12 *Tfeb^F/F^* eyes. *n* = 20 *Olig2-Cre; Tfeb^F/F^* eyes. (*E*) The TFEB–autophagy axis acts in the newly formed oligodendrocytes to remodel myelin membrane and reduce myelin whorls, thereby regulating myelin integrity. During axon ensheathment, newly formed oligodendrocytes dramatically expand the myelin membrane to provide maximal coverage for axons. Myelin abnormalities, such as myelin whorls, occur along newly generated myelin sheaths. TFEB binds directly to autophagy genes and activates autophagy flux, thereby limiting myelin membrane protrusions and whorls. Created in BioRender. L. Sun (2026) https://BioRender.com/id3onr3. Error bars indicate SEM. Open circles in *C* and *D* represent the mean value. Two-way ANOVA with Sidak’s multiple comparisons test for *C* and *D*.

To assess whether the visual function of *Tfeb* cKO mice is impaired at the peak of myelin whorl presence, we employed the visual evoked potential (VEP) assay, a postretinal measure of electrical responses recorded in the visual cortex evoked by visual stimuli at different light intensities. Both N1 amplitudes and latencies from VEPs were recorded from P14 *Tfeb* cKO mice and their littermate controls after overnight dark adaptation (Fig. 7*A*).

As expected, both control and *Tfeb* cKO mice exhibited light-intensity-dependent reductions in N1 latency, with the longest N1 latency recorded at 0.01 cd s/m^2^ (Fig. 7*C*). While no significant difference in N1 amplitudes was observed between control and *Tfeb* cKO mice (Fig. 7*D*), *Tfeb* cKO mice exhibited significantly prolonged VEP latencies at both 0.1 and 0.5 cd s/m^2^, as well as an overall significantly altered N1 latency curve compared to controls (Fig. 7*C*). Notably, at the light intensity of 0.5 cd s/m^2^ *Tfeb* cKO exhibited an average of 10 milliseconds delay compared to controls (Fig. 7*B*; 70.04 ± 1.99 ms for controls and 79.55 ± 1.84 ms for *Tfeb* cKO, approximately 14% increase in *Tfeb* cKO; *n* = 12 eyes for control, *n* = 20 eyes for *Tfeb* cKO). We observed a similar VEP phenotype in P14 *Atg5* cKO mice (*SI Appendix*, Fig. S6 *D* and *E*), which phenocopy *Tfeb* cKO mice with respect to whorl density but do not exhibit hypermyelination (Fig. 6 and *SI Appendix*, Fig. S5). Importantly, VEP N1 latency delays observed in *Tfeb* cKO mice were not due to abnormalities in nodes of Ranvier (NoR), which can substantially slow visual signal transduction (24). This was supported by the findings that the density of Caspr^+^ AnkG^+^ double-positive NoR was unchanged in *Tfeb* cKO optic nerves compared to controls (*SI Appendix*, Fig. S6*A*; quantified in *SI Appendix*, Fig. S6*B*) and that *Tfeb* cKO optic nerves exhibited normal paranodal ultrastructure (*SI Appendix*, Fig. S6*C*). Thus, P14 *Tfeb* cKO optic nerves have normal NoR density and intact paranodal structure but exhibit a significant delay in visual signal transduction from the retina to the visual cortex. Taken together, our studies show that disruption to TFEB signaling and autophagy in newly formed oligodendrocytes leads to aberrant generation of CNS myelin during rapid axon ensheathment (Fig. 7*E*).

## Discussion

As an essential part of the nervous system, myelin undergoes profound remodeling during its establishment, maintenance, and repair following injury (4, 25–27). In searching for proper axons for myelination, newly formed internodes can extend and retract multiple times before stabilizing (4, 26, 27). The newly developed *Enpp6-IRES-CreER^T2^* knock-in mouse line, specifically expressed in transient premyelinating oligodendrocytes, allowed us to investigate early stages of myelin ensheathment in vivo (20). Using this tool and volume EM, we found that the disruption to TFEB signaling and autophagy in newly formed oligodendrocytes leads to aberrant generation of CNS myelin at the onset of axon ensheathment. This period coincides with rapid myelin membrane expansion and extensive myelin coverage for axons. Importantly, the increased abundance of myelin membrane protrusions and myelin whorls observed in both *Tfeb* cKO and *Atg5* cKO optic nerves does not result from increased oligodendrocyte numbers or excessive myelin wrapping, indicating a distinct mechanism governing myelin integrity.

Both *Tfeb* and *Atg5* cKO optic nerves exhibit elevated myelin membrane protrusions (revealed by MBP immunostaining and genetic labeling) and myelin whorls (revealed by TEM and SBEM) during development. Both structures are tightly associated with myelinated axons and are encapsulated by MBP^+^ myelin sheaths. Moreover, both structures exhibit both singular and rosette-like/clustered features. Intriguingly, the density of MBP^+^ myelin protrusions is comparable to that of myelin whorls, while their sizes appear larger than those of whorls. Although these two structures are highly correlated, there is no evidence yet demonstrating that membrane protrusions develop into whorls. Future experiments using advanced EM imaging techniques, such as correlative light and electron microscopy (CLEM), as well as live imaging of myelin sheaths, will be needed to determine their spatiotemporal relationship during axon ensheathment.

Although our genetic evidence strongly implicates autophagy in restricting myelin whorl abundance, our current data do not directly visualize autophagic structures engulfing myelin whorls. Therefore, we cannot exclude the possibility that autophagy regulates whorl abundance indirectly through broader effects on membrane homeostasis in oligodendrocytes. Nevertheless, our observations that myelin whorls contain multilayered myelin and protrude from newly generated myelin sheaths raise the possibility: Myelin membranes produced during rapid axon ensheathment may serve as substrates for autophagy. This may occur through an endocytosis-dependent mechanism, in which parts of newly formed myelin sheaths are endocytosed and fuse with autophagosomes to become amphisomes, leading to myelin degradation in autolysosomes (28). Indeed, a recent study showed that autophagosomes form within the myelin sheaths and that myelin proteins such as MBP, MOG, and MAG are present in oligodendrocyte autophagosomes (29). In addition, the endoplasmic reticulum (ER), a major source of the phagophore that initiates autophagy, is found abundantly in the inner tongue of myelin sheaths (30), where myelin whorls are also observed in our study (Fig. 2 and *SI Appendix*, Fig. S2). Future studies using CLEM and live imaging approaches will be necessary to directly visualize the interaction between myelin whorls and the autophagy machinery in oligodendrocytes.

During development, myelin debris is partially cleared by microglia, which actively phagocytose MBP^+^ fragments and even entire myelin sheaths (4, 13, 31). Consistent with these observations, we identify subsets of myelin whorls that are not associated with myelinated axons, which may be shed into the extracellular space or readily engulfed by microglia. A recent study showed that *Csf1r^DM^* mutant zebrafish, which harbor ~5% of microglia and macrophages presented in controls, exhibit a significantly increased number of myelin outfoldings and fragments (4). We found that detached whorls are increased in *Tfeb* cKO optic nerves, whereas the density of myelin outfoldings remains unchanged, suggesting that oligodendrocytes and microglia may play complementary roles in limiting myelin abnormalities. Together, these observations support a model in which TFEB-directed autophagy can be initiated locally within myelin sheaths to reduce myelin whorls, while phagocytes such as microglia engulf and eliminate myelin whorls shed into the extracellular space, as well as other myelin abnormalities. It will be of great interest to investigate whether and to what extent oligodendrocyte TFEB signaling cooperates with microglia to safeguard myelin integrity.

In the brain, *Tfeb* cKO mice lack premyelinating oligodendrocyte cell death and exhibit significantly increased oligodendrocyte numbers (15, 18). Surprisingly, the optic nerves of *Tfeb* cKO mice exhibit normal oligodendrocyte lineage cell numbers. This is not because oligodendrocytes do not undergo apoptosis in the optic nerve, as *Olig2-Cre; Bax^F/F^; Bak^−/−^* optic nerves exhibit significantly elevated oligodendrocyte numbers (*SI Appendix*, Fig. S3). Therefore, TFEB is either not required for, or is functionally redundant in, optic nerve oligodendrocyte apoptosis. However, our study reveals that TFEB has a function in the robust activation of autophagy in optic nerve oligodendrocytes to promote myelin membrane remodeling and whorl reduction. This process is autophagy-dependent but independent of TFEB’s repressive role in myelination, as *Atg5* cKO optic nerves phenocopy *Tfeb* cKO optic nerves with excessive myelin whorls but no hypermyelination.

Compacted and properly formed myelin sheaths are essential for proper nerve signal conduction in the CNS (32). Abnormal myelin, such as uncompacted myelin due to the deletion of MBP in *Shiverer* mice, leads to motor malfunctions, seizures, and delayed nerve pulse propagation (33). Both *Tfeb* cKO and *Atg5* cKO mice exhibit the VEP phenotype at the peak presence of myelin whorl abnormalities; however, the causal relationship between excessive myelin whorls and prolonged VEP latency remains unclear and requires further investigation. This question remains challenging to address due to the limited tools available to specifically alter myelin whorl abundance.

Taken together, our findings identify a role for TFEB-dependent autophagy in establishing proper myelin structure during development, providing insights into the oligodendrocyte-intrinsic mechanisms that regulate myelin integrity.

## Materials and Methods

### Animals.

All experiments conducted with animals followed protocols approved by the Institutional Animal Care and Use Committee (IACUC) of University of Texas Southwestern Medical Center and the Guide for Care and Use of Laboratory Animals. The mice were housed in a 12:12 light–dark cycle with water and food available ad libitum. The date of birth in this study was designated as postnatal day 0 (P0) for all experiments. Both males and females were used for all experiments.

Mouse lines used in this study include *Tfeb^LacZ^* (15), *Olig2-Cre* (Jackson Laboratory, B6.129-*Olig2tm1.1(cre)Wdr/J*, stock#025567), *Tfeb^Flox^* (15), *Enpp6-IRES-CreER^T2^* (20), *mT/mG* (Jackson Laboratory, Gt(ROSA)26Sortm4(ACTB-tdTomato, -EGFP)Luo/J, stock#007576), *Cnp-Cre* (34), *Bax^Flox^; Bak^-^* (Jackson Laboratory, B6;129-*Baxtm2Sjk Bak1tm1Thsn/J*, stock #006329), and *Atg5^Flox^* (RIKEN, *Atg5^tm1Myok^*, stock#RBRC02975).

Experimental procedures are described in *SI Appendix*, *Materials* and *Methods*.

## Supplementary Material

Appendix 01 (PDF)

Movie S1. A video showing 767 consecutive SBEM sections from a P14 *Tfeb^F/F^* optic nerve, with myelin whorls annotated by different colors. The video is played at 7 SBEM sections per second. Scale bar: 10 μm.

Movie S2. A 3D rendering video showing annotated whorls from a P14 *Tfeb^F/F^*optic nerve. Scale bar: 10 μm.

Movie S3. A video showing 473 consecutive SBEM sections from a P14 *Olig2-Cre*; *Tfeb^F/F^* optic nerve, with whorls annotated by different colors. The video is played at 7 SBEM sections per second. Scale bar: 10 μm.

Movie S4. A 3D rendering video showing annotated whorls from a P14 *Olig2-Cre; Tfeb^F/F^* optic nerve. Scale bar: 10 μm.

Movie S5. Consecutive SBEM sections and annotation of a “detached” whorl in a P14 *Tfeb^F/F^* optic nerve. The video is played at 4 SBEM sections per second. Scale bar: 1 μm.

Movie S6. Consecutive SBEM sections and annotation of a whorl associated with a myelinated axon in a P14 *Tfeb^F/F^* optic nerve. The video is played at 4 SBEM sections per second. Scale bar: 1 μm.

Movie S7. Consecutive SBEM sections and annotation of a “detached” whorl in a P14 *Olig2-Cre; Tfeb^F/F^* optic nerve. The video is played at 4 SBEM sections per second. Scale bar: 1 μm.

Movie S8. Consecutive SBEM sections and annotation of a whorl associated with a myelinated axon in a P14 *Olig2-Cre; Tfeb^F/F^* optic nerve. The video is played at 4 SBEM sections per second. Scale bar: 1 μm.

Movie S9. 3D rendering of MBP^+^ processes in P7 *Tfeb^F/F^* optic nerve, showing the smooth MBP^+^ internode. See also Figure 3D.

Movie S10. 3D rendering of MBP^+^ processes in P7 *Tfeb^F/F^* optic nerve, showing several MBP^+^ protrusions along the internode. See also Figure 3D.

Movie S11. 3D rendering of MBP^+^ processes in P7 *Tfeb^F/F^* optic nerve, showing an MBP^+^ rosette structure formed along the internode. See also Figure 3D.

## Data Availability

Study data are included in the article and/or supporting information.
