# Supplementary material for: Disruption to TFEB signaling and autophagy in newly formed oligodendrocytes leads to aberrant generation of CNS myelin
Source: Proc Natl Acad Sci U S A. 2026 Jun 10;123(24):e2528668123. doi: 10.1073/pnas.2528668123 (PMC13273360; doi:10.1073/pnas.2528668123)
Supplement: Supplementary file 1 — Appendix 01 (PDF) [file pnas.2528668123.sapp.pdf]

Supporting Information for

# Disruption to TFEB Signaling and Autophagy in Newly Formed Oligodendrocytes Leads to Aberrant Generation of CNS Myelin

Daniela Barbosa, Aksheev Bhambri, Miguel Vasquez, Yihe Zhang, Gabrielle Sanchez, Katherine J. Wert, Natalia V Gounko, Mark H. Ellisman, Lu O. Sun

Corresponding Author: Lu O. Sun
**Email:** lu.sun@utsouthwestern.edu (L.O.S)

**This PDF file includes:**

Supporting text
Figures S1 to S6
Legends for Movies S1 to S11
SI References

**Other supporting materials for this manuscript include the following:**

Movies S1 to S11

## Materials and Methods

### X-gal staining

*Tfeb*$^{LacZ/+}$ mice at P14 were perfused with freshly made chilled phosphate-buffered saline (PBS) (Sigma-Aldrich, Cat#P3813-10PAK) and 4% paraformaldehyde (PFA) (Fisher Scientific, Cat#50-980-489) solutions. The optic nerves were micro-dissected and fixed for 1 hour at 4°C, then transferred to a 30% sucrose solution (Sigma-Aldrich, Cat#S9378-1KG) in 1X PBS at 4°C overnight. Optic nerves sections (12 µm) were rinsed twice in PBS before X-gal reaction. After rinsing, optic nerve sections were incubated with the X-gal staining solution containing 5 mM potassium ferricyanide (Sigma-Aldrich, Cat#702587), 5 mM potassium ferrocyanide (Sigma-Aldrich, Cat#31254), 2 mM $MgCl_2$ (Sigma-Aldrich, Cat#M8266), and 1 mg/mL X-gal (Sigma-Aldrich, Cat#10651745001) for 24 hours at 37°C in a humified chamber. Sections were rinsed three times with PBS and imaged with a Keyence BZ-810 all-in-one microscope.

### *In situ* hybridization

*In situ* hybridization was performed on fresh frozen P7 and P14 wild-type optic nerve sections (12 µm), using the RNAscope Multiplex Fluorescent Detection kit (ACD Bio, Cat#323100). The optic nerves were stained with *Tfeb* probe (ACDBio, Cat#434701), *Olig2* probe (ACD Bio, Cat#447091-C2), and *Enpp6* probe (ACDBio, Cat#511021-C2), paired with the corresponding TSA vivid fluorescent dyes (ACDbio, Cat#323270). Fluorescent images were taken with a Zeiss LSM 700 inverted confocal microscope.

### Immunostaining

Optic nerves collected from P7 pups were dissected without perfusion. For optic nerves collected on or after P14, mice were perfused with PBS and 4% PFA/PBS solutions. The optic nerves were fixed overnight in 4% PFA/PBS and then transferred to a 30% sucrose/PBS solution at 4°C overnight. Optic nerves were sectioned at 30-µm thickness at P5, at 20-µm thickness at P7, and at 12-µm thickness at P14. Sections were rinsed with blocking solution containing 0.1% - 0.5% Triton X-100 (PBST; Sigma-Aldrich, Cat#T8787) and 10% normal goat or donkey serum for 15 minutes at room temperature. The sections were incubated overnight at 4°C in the primary antibody solution. Primary antibodies used in this study include: Mouse anti-CC1 (Abcam, 1:100, Cat#AB16794), Rabbit anti-Olig2 (Millipore, 1:200, Cat#AB9610), Rabbit anti-PDGFRα (Rabbit anti-PDGFRα (Abcam, 1:200, Cat#AB203491), Rat anti-MBP (Abcam, 1:200, Cat#AB7349), Rabbit anti-p62 (Proteintech, 1:200, Cat#18420-1-AP), Guinea Pig anti-CASPR (Gift from the Bhat Lab, 1:500), and Mouse anti-Ank-G (Antibodies Inc., 1:200, Cat#75-146). Optic nerve sections were rinsed at room temperature three times for 10 minutes with PBST solution. Sections were incubated with AlexaFluor 488-, 555-, 594-, or 647-conjugated secondary antibodies (ThermoFisher, 1:1000) and DAPI (ThermoFisher, 5 µg/mL, Cat#62247), diluted in PBST containing 2% normal goat or donkey serum for one hour at room temperature. The sections were washed with PBST for 10 minutes, 4 times, air-dried for 5 minutes, and mounted using Vectashield Hardset Antifade Mounting Medium (Vector Laboratories, Cat#H-1700-10). Fluorescent images were taken with the Nikon A1-SIM-E or a Zeiss LSM 700 confocal microscope.

### 4-hydroxytamoxifen (4HT) and tamoxifen injections

P4 *Enpp6-IRES-CreER*$^{T2}$*; mT/mG* pups were intraperitoneally injected with 4HT (Sigma-Aldrich, Cat#H7904-25mg) at a concentration of 5 mg/mL, collected at P7 or P14. *Enpp6-IRES-CreER*$^{T2}$*; mT/mG* and *Enpp6-IRES-CreER*$^{T2}$*; Tfeb*$^{F/F}$*; mT/mG* pups were

intraperitoneally injected with 20 µL of tamoxifen (10 mg/mL; Sigma-Aldrich, Cat#T5648) at P4 and P5, and the optic nerve was collected at P14.

**Transmission electron microscopy (TEM)**
Animals at or older than P14 were perfused with chilled EM fixative containing 4% paraformaldehyde, 2.5% glutaraldehyde, and 0.2% picric acid in 0.01M phosphate buffer. Optic nerves were micro-dissected and kept at 4°C before submission to the UT Southwestern EM core. After five rinses in 0.1 M sodium cacodylate buffer, the tissues were post-fixed twice in 1% osmium tetroxide with 1.5% $K_3[Fe(CN)_6]$ in 0.1 M sodium cacodylate buffer for 1 hour each at room temperature. Tissues were rinsed with water and *en bloc* stained with 0.5% aqueous uranyl acetate in 25% methanol overnight at 4°C. After five rinses with water, specimens were stained with 0.02M lead nitrate in 0.03M L-aspartate for 30 minutes at 60°C. Samples were dehydrated with increasing concentration of ethanol and infiltrated with Embed-812 resin and polymerized in a 60°C oven overnight. Blocks were sectioned with a diamond knife (Diatome) on a Leica Ultracut UCT ultramicrotome (Leica Microsystems) and collected onto formvar-coated copper grids. Images were acquired on a JEOL JEM-1400 Plus TEM operated at 80 kV using an AMT-BioSprint 16M CCD camera. Grids were imaged at 800X, 1000X, and 5000X magnifications for quantifications and representative images.

**Serial block-face tissue processing and imaging**
Intact optic nerves were washed in cold 0.15M sodium cacodylate buffer with 2 mM $CaCl_2$, pH7.4 ("cacodylate buffer", Sigma Aldrich) on ice. The optic nerves were then incubated in a 2% osmium tetroxide (EMS), 1.5% potassium ferrocyanide solution (J.T. Baker Chemical Co.) in cacodylate buffer. Samples were washed with cacodylate buffer, then incubated in filtered 0.5% aqueous thiocarbohydrazine (TCI America), followed by double-distilled water washes and then incubation in 2% aqueous osmium tetroxide. Samples were washed with water, then incubated cold in filtered 2% aqueous uranyl acetate (EMS), followed by water washes, incubation in warmed, freshly made filtered lead aspartate, and then washed with water.

Samples were dehydrated in a cold ethanol (Koptec) gradient series: 20%, 50%, 70%, 90%, and 100% on ice, followed by dehydration in 100% ethanol twice at room temperature, then 100% dry by acetone (Fisher Scientific). Samples were then infiltrated in a gradient series: 25% Durcupan ACM resin (Sigma-Aldrich): 75% dry acetone, then 50% resin: 50% dry acetone, then 75% resin: 25% dry acetone, and finally 100% resin. Durcupan ACM resin was made by mixing 11.4 g component A, 10 g component B, 0.3 g component C, and 0.1 g component D. Samples were embedded and polymerized in 100% Durcupan ACM resin at 60°C for 48 hours. Samples were cut and trimmed to the region of interest, using micro-CT scans for precision adjustments.

Sample image volumes were collected on a Gemini 450 field emission SEM (Carl Zeiss Microscopy) with a 3View stage (Gatan Inc.) at a 2.5kV accelerating voltage, 1µs pixel dwell time, 7.0 nm pixel size, 60 nm section thickness, with a raster size of 20k x 10k at high vacuum and focal gas injection-based charge compensation.

**Serial block-face scanning electron microscopy (SBEM) image analysis**
The SBEM Z-stack data were analyzed using the 3DMOD software. Whorls were manually annotated by creating new objects and utilizing the software's sculpting and interpolation tools for outlining. Each object was delineated across every Z-plane where it was present, and the contour was adjusted appropriately using the sculpting tool. After annotating and meshing all objects, the volume and count of the objects were extracted from the software. To calculate the percent volume of whorls within the full SBEM S-stack data for control

and *Tfeb* cKO, we summed the total whorl volumes of all objects and divided by the total volume of the SBEM Z-stack. Individual whorl size was directly extracted from the 3DMOD software after all objects were meshed, and we took the 'Total Volume Inside Mesh' since it is the most accurate. The Total Volume Inside Mesh represents the accurate volume from the interpolated mesh of the object created.

**Immuno-EM labeling and imaging**

For pre-embedding myelin basic protein (MBP) labeling, P7 optic nerves were fixed overnight at 4 °C using a solution containing 2% paraformaldehyde (PFA, EMS, Cat#15714) and 0.01% glutaraldehyde (GA, EMS, Cat#16210) in 0.1 M Phosphate Buffer (PB) (pH7.4). Subsequently, samples were washed with 0.1 M PB, followed by blocking in 0.1% sodium borohydride (Sigma-Aldrich, Cat#71320-25g) in 0.1 M PB for 30 min on ice. The samples were washed again with 0.1 M PB, followed by a second blocking step in 0.01% glycine (Sigma-Aldrich, Cat#L-5626), 0.01% lysine (Sigma-Aldrich, Cat#G7126-100g), 0.1% cold water fish gelatin (CWFG, EMS, Code#900.033, Lot#18093), 20% BSA (Sigma-Aldrich, Cat#A7030-10G), 10% goat serum (Vector Laboratories, Cat#S-1000-20) and 0.01% Triton X (EMS, Cat#22146) in 0.1 M PB for 2 hours. Afterwards, some sections were incubated with primary antibody (1:200, rat anti-MBP aa82-87, Cat# MCA409S) overnight at 4 °C. The next day, samples were washed with 0.1 M PB and incubated with secondary biotin-conjugated antibody (1:200, goat anti-Rat IgG, Biotinylated; Vector Laboratories, Cat#BA-9400-1.5) in the same incubation buffer for 2 hours at room temperature.

To visualize DAB/GSSP immunoreactivity, we used the avidin–biotin complex (ABC) method. The ABC complex is prepared strictly in accordance with the manufacturer's instructions (Vector Laboratories, Cat#PK-6100). The initial steps of the protocol involve a series of washing steps: two cycles of immersion in 0.1 M PB, followed by three immersions in PB containing 0.01% Triton X-100 for 10 min each on ice. Afterwards, samples were incubated with the ABC complex over the course of 1 hour at room temperature. To visualize the immunoreactivity, we incubated the samples with a solution containing 5 mg of 3.3′-DAB (Sigma-Aldrich, Cat#SLBQO700V-10mg) and 0.03% hydrogen peroxide dissolved in 10 ml of 0.1 M PB solution for 10 min at room temperature. The termination of the reaction was induced by immersing the samples in cold 0.1 M PB, whereafter the samples were fixed with 2% GA in 0.1 M PB for 1 hour, on ice. Following this, samples were subsequently washed four cycles in 0.1 M PB, followed by three cycles in 2% sodium acetate (EMS) diluted in distilled water for 10 minutes each on ice. Samples were stored for 5 days in 10% sodium thioglycolate in distilled water (Sigma-Aldrich) and maintained at a temperature of 4 °C. The labeling was enhanced by the implementation of the GSSP method (1). Post GSSP reaction, the samples were washed in 0.1 M sodium cacodylate (EMS) buffer for four 10-min cycles on ice. The samples were subjected to staining with 1% osmium tetroxide (EMS) and 1.5% potassium ferrocyanide (EMS) in 0.1 M sodium cacodylate trihydrate (EMS) buffer for 20 min. This was followed by five sequential washing steps in distilled water, each for 7 min. Subsequently to the washes, sections underwent a dehydration process involving ascending ethanol solution steps (30%, 50%, 70%, 80%, 95%), 10 min per step, at 4 °C. Additionally, the samples were subjected for two 15-min treatments, each with absolute ethanol at room temperature and infiltrated with medium Epon 812/ethanol mixtures overnight. The following day, samples were flat-embedded in Epon 812 (EMS) between two microscopic slides and ACLAR film (EMS), and subsequently polymerized for 2 days at 60 °C. Next, 70 nm sections were cut using a Leica Ultracut UCT ultramicrotome (Leica Microsystems) and the sections were collected onto a formvar film-coated 100-mesh copper grid (EMS). Finally, the sections were counterstained with uranyl acetate and lead citrate. TEM images were captured

using a JEOL JEM-1400 Plus TEM operated at 80 kV using an AMT-BioSprint 16M CCD camera.

**Visual behavior assays**

Behavioral studies were conducted blindly, with no knowledge of the animals' genotypes. Mice were dark-adapted overnight for at least 12 hours. All mouse handling during the study was performed under dim red-light illumination. Dark-adapted visual evoked potentials (VEPs) were recorded using the Celeris Rodent Electroretinography system (Diagnosys LLC). Pupils were dilated with topical 2.5% phenylephrine hydrochloride and 1% tropicamide. Mice were sedated via intraperitoneal injection of ketamine/xylazine in 1X PBS at a dose of 0.1 ml/10 g body weight of anesthesia [1 ml of ketamine (100 mg/ml) and 0.1 ml of xylazine (20 mg/ml) in 8.9 ml of 1X phosphate-buffered saline (PBS)]. The body temperature was maintained at 37°C throughout the VEP procedure, and eyes were kept moist with a layer of 0.3% Hypromellose gel (GenTeal, Alcom). Scotopic VEP recordings were performed using a series of 20 repetitive light flashes at intensities of 0.01, 0.1, 0.5, 1.0, 2.0, and 3.0 cd·s/$m^2$. Visual responses from these 20 repetitive light flashes were averaged into a single curve for each light intensity in each animal. Following the scotopic VEP recordings, mice were allowed to recover on a 37°C warming surface until fully conscious.

**Imaris 3D rendering**

Confocal images of sparsely labeled *mT/mG* oligodendrocytes were subjected to 3D rendering using the Imaris software (Filament Tracer module). Representative images were reconstructed using manual tracing and automated filament thickness measurements based on confocal signal intensity for accurate visualization.

**Quantification and statistical analysis**

Data analyses in this study were performed using GraphPad Prism 10. Graphs are presented as mean ± SEM, and the sample sizes "n" are outlined in the text and figure legends. Each data point represents a single animal. Statistical significance was assessed using a two-tailed, unpaired Student's *t*-test when comparing two independent groups. For comparisons involving multiple groups, one-way or two-way ANOVA, followed by Tukey's or Sidak's multiple comparisons test, was performed. A p-value of $< 0.05$ was considered statistically significant.

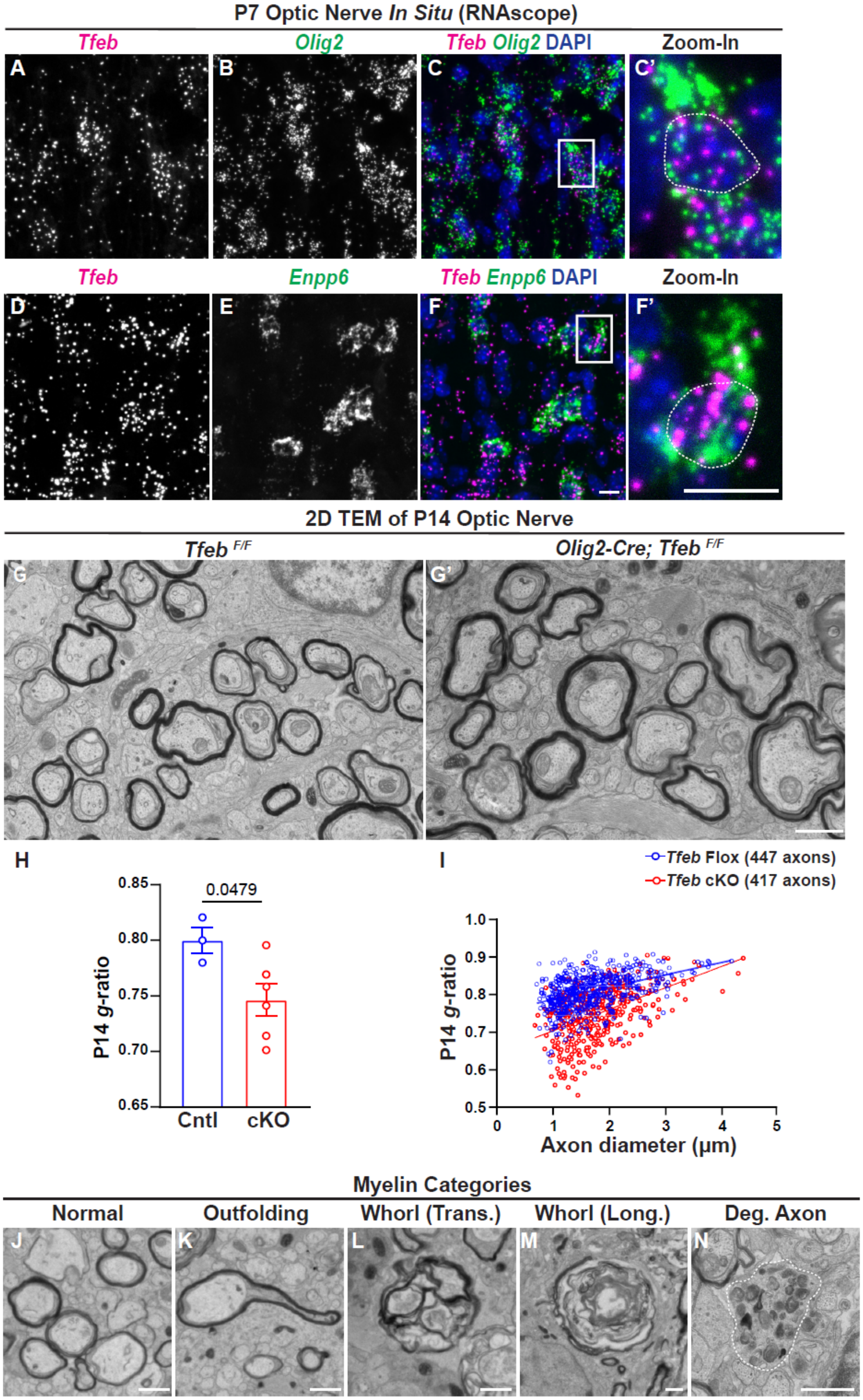

P7 Optic Nerve In Situ (RNAscope)
Tfeb
Olig2
Tfeb Olig2 DAPI
Zoom-In
A
B
C
C'
Tfeb
Enpp6
Tfeb Enpp6 DAPI
Zoom-In
D
E
F
F'
2D TEM of P14 Optic Nerve
Tfeb F/F
Olig2-Cre; Tfeb F/F
G
G'
H
0.0479
P14 g-ratio
0.85
0.80
0.75
0.70
0.65
Cntl
cKO
I
Tfeb Flox (447 axons)
Tfeb cKO (417 axons)
P14 g-ratio
1.0
0.9
0.8
0.7
0.6
0.5
0
1
2
3
4
5
Axon diameter (μm)
Myelin Categories
Normal
Outfolding
Whorl (Trans.)
Whorl (Long.)
Deg. Axon
J
K
L
M
N

**Fig. S1. Characterization of *Tfeb* expression, myelin sheath thickness, and myelin abnormalities in *Tfeb* conditional knockout (cKO) optic nerves.**

**(A–F')** Double fluorescent *in situ* hybridization using probes against *Tfeb* (magenta in A, C, C', D, F, and F'), *Olig2* (green in B, C, and C'), and *Enpp6* (green in E, F, and F') on P7 optic nerves. (C') and (F') represent enlarged views of insets in (C) and (F), respectively. Dashed lines in (C') and (F') demarcate $Tfeb^{+}$ $Olig2^{+}$ and $Tfeb^{+}$ $Enpp6^{+}$ cells, respectively.

**(G and G')** Representative transverse TEM micrographs of P14 $Tfeb^{F/F}$ (G) and *Olig2-Cre;* $Tfeb^{F/F}$ optic nerves (G').

**(H and I)** Quantification of average *g*-ratio (H) and a scatter plot showing *g*-ratios of myelinated axons as a function of axon diameter in P14 $Tfeb^{F/F}$ (*Tfeb* Flox: Cntl) and *Olig2-Cre;* $Tfeb^{F/F}$ (*Tfeb* cKO: cKO) mice (I).

**(J–N)** Representative TEM micrographs of normal myelin (J), myelin outfolding (K), a myelin whorl on the transverse section (L), a myelin whorl on the longitudinal section (M), and a degenerative axon (N) observed in P14 wild-type optic nerves. Note that degenerate axons are filled with electron-dense organelles such as lysosomes and autophagosomes (N), which are distinct from myelin whorls (L and M).

Error bars represent SEM. Open circles in (H) represent individual animals. Open circles in (I) represent individual axons. Two-tailed Student's *t*-test for (H). Scale bars: 10 µm in (F) for (A), (B), (C), (D), (E), and (F); 10 µm in (F') for (C') and (F'); 1 µm in (G') for (G) and (G'); and 1 µm in (J)–(N).

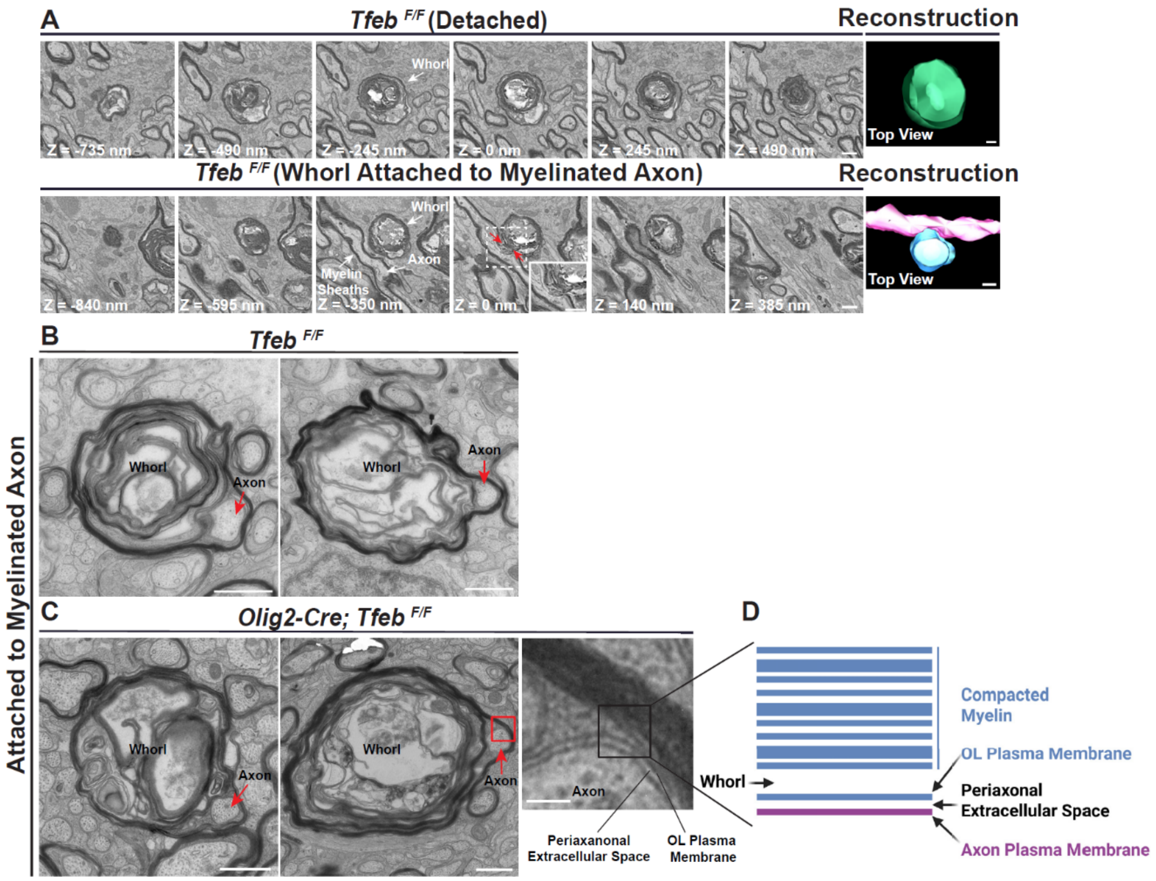


**Fig. S2. SBEM and TEM reveal the geometry of detached myelin whorls and those associated with myelinated axons.**

**(A)** Representative SBEM sections along the z-axis and reconstruction of a "detached" whorl (top row) and a whorl associated with a myelinated axon (bottom row) in the *Tfeb*$^{F/F}$ optic nerve. White boxes and red arrows highlight the connection between the "attached" whorl and the myelinated axon.

**(B and C)** Representative TEM micrographs of whorls attached to myelinated axons in P14 *Tfeb*$^{F/F}$ (B) and *Olig2-Cre; Tfeb*$^{F/F}$ optic nerves (C). Red arrows indicate axons. The red box indicates the zoomed-in region shown on the rightmost column. Axon, periaxonal extracellular space, and oligodendrocyte (OL) plasma membrane are indicated.

**(D)** Schematic depicting the spatial relationship among the axon plasma membrane, periaxonal extracellular space, OL plasma membrane, whorl, and compacted myelin sheaths based on TEM and SBEM data. Created in BioRender. Sun, L. (2026) https://BioRender.com/id3onr3.

Scale bars: 1 μm in (A), insets of (A), (B), and (C); and 100 nm in the zoom-in micrograph of (C).

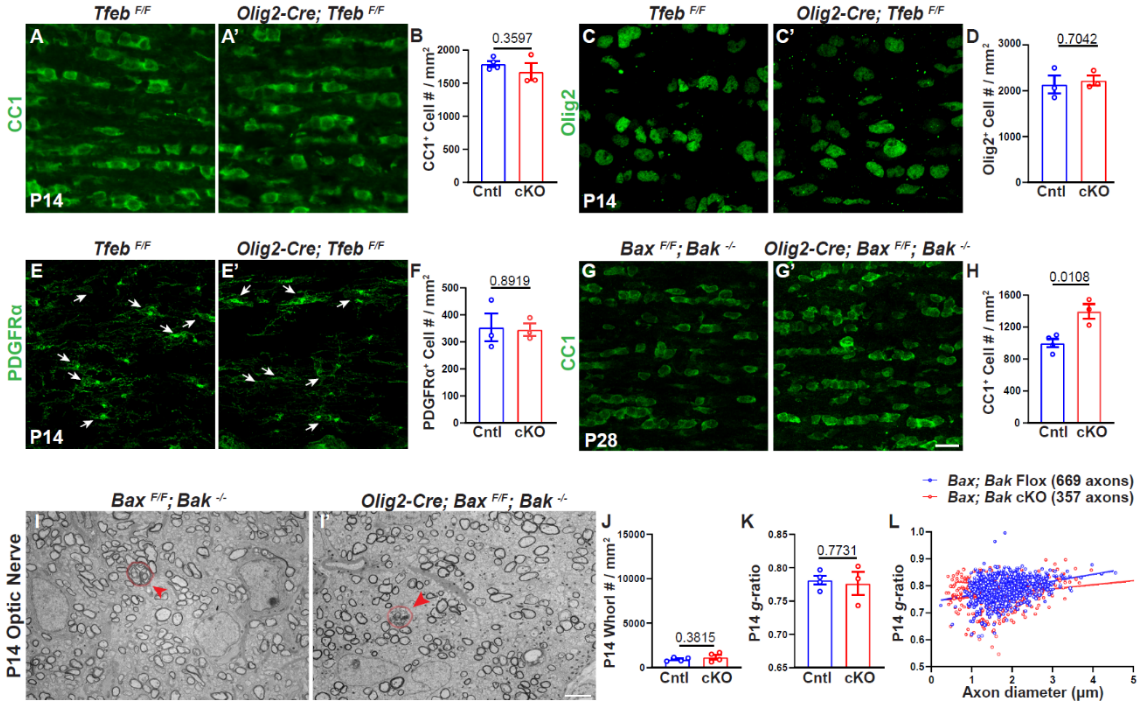


**Fig. S3. Genetic blockade of oligodendrocyte apoptosis does not increase myelin whorl density or myelin sheath thickness.**

**(A and A')** Representative confocal micrographs of P14 *Tfeb*$^{F/F}$ (A) and *Olig2-Cre; Tfeb*$^{F/F}$ (A') optic nerve sections stained with a CC1 antibody.

**(B)** Quantification of CC1$^{+}$ cell density in P14 *Tfeb*$^{F/F}$ (Cntl) and *Olig2-Cre; Tfeb*$^{F/F}$ (cKO) optic nerves.

**(C and C')** Representative images of P14 *Tfeb*$^{F/F}$ (C) and *Olig2-Cre; Tfeb*$^{F/F}$ (C') optic nerve sections stained with an antibody raised against Olig2.

**(D)** Quantification of Olig2$^{+}$ cell density in P14 *Tfeb*$^{F/F}$ (Cntl) and *Olig2-Cre; Tfeb*$^{F/F}$ (cKO) optic nerves.

**(E and E')** Representative images of P14 *Tfeb*$^{F/F}$ (E) and *Olig2-Cre; Tfeb*$^{F/F}$ (E') optic nerve sections stained with a PDGFRα antibody. White arrows indicate PDGFRα$^{+}$ cell bodies.

**(F)** Quantification of PDGFRα$^{+}$ cell density in P14 *Tfeb*$^{F/F}$ (Cntl) and *Olig2-Cre; Tfeb*$^{F/F}$ (cKO) optic nerves.

**(G and G')** Representative images of P28 *Bax*$^{F/F}$*; Bak*$^{-/-}$ (G) and *Olig2-Cre; Bax*$^{F/F}$*; Bak*$^{-/-}$ optic nerve sections (G') stained with a CC1 antibody.

**(H)** Quantification of CC1$^{+}$ cell density in P28 *Bax*$^{F/F}$*; Bak*$^{-/-}$ (Cntl) and *Olig2-Cre; Bax*$^{F/F}$*; Bak*$^{-/-}$ (cKO) optic nerves.

**(I and I')** Representative transverse TEM micrographs of P14 *Bax*$^{F/F}$*; Bak*$^{-/-}$ (I) and *Olig2-Cre; Bax*$^{F/F}$*; Bak*$^{-/-}$ (I') optic nerves. Red arrows indicate whorls (pseudo-colored red).

**(J)** Quantification of whorl density in P14 *Bax*$^{F/F}$*; Bak*$^{-/-}$ (Cntl) and *Olig2-Cre; Bax*$^{F/F}$*; Bak*$^{-/-}$ (cKO) optic nerves using transverse TEM sections.

**(K and L)** Quantification of average *g*-ratio (K) and a scatter plot showing *g*-ratios of myelinated axons as a function of axon diameter (L) in P14 *Bax*$^{F/F}$*; Bak*$^{-/-}$ (Cntl) and *Olig2-Cre; Bax*$^{F/F}$*; Bak*$^{-/-}$ (cKO) optic nerves, using transverse TEM sections.

Error bars indicate SEM. Open circles in (B), (D), (F), (H), (J), and (K) represent individual animals. Open circles in (L) represent individual axons. Two-tailed Student's *t*-test for (B),

(D), (F), (H), (J), and (K). Scale bars: 20 μm in (G’) for (A), (A’), (C), (C’), (E), (E’), (G), and (G’); and 4 μm in (I’) for (I) and (I’).

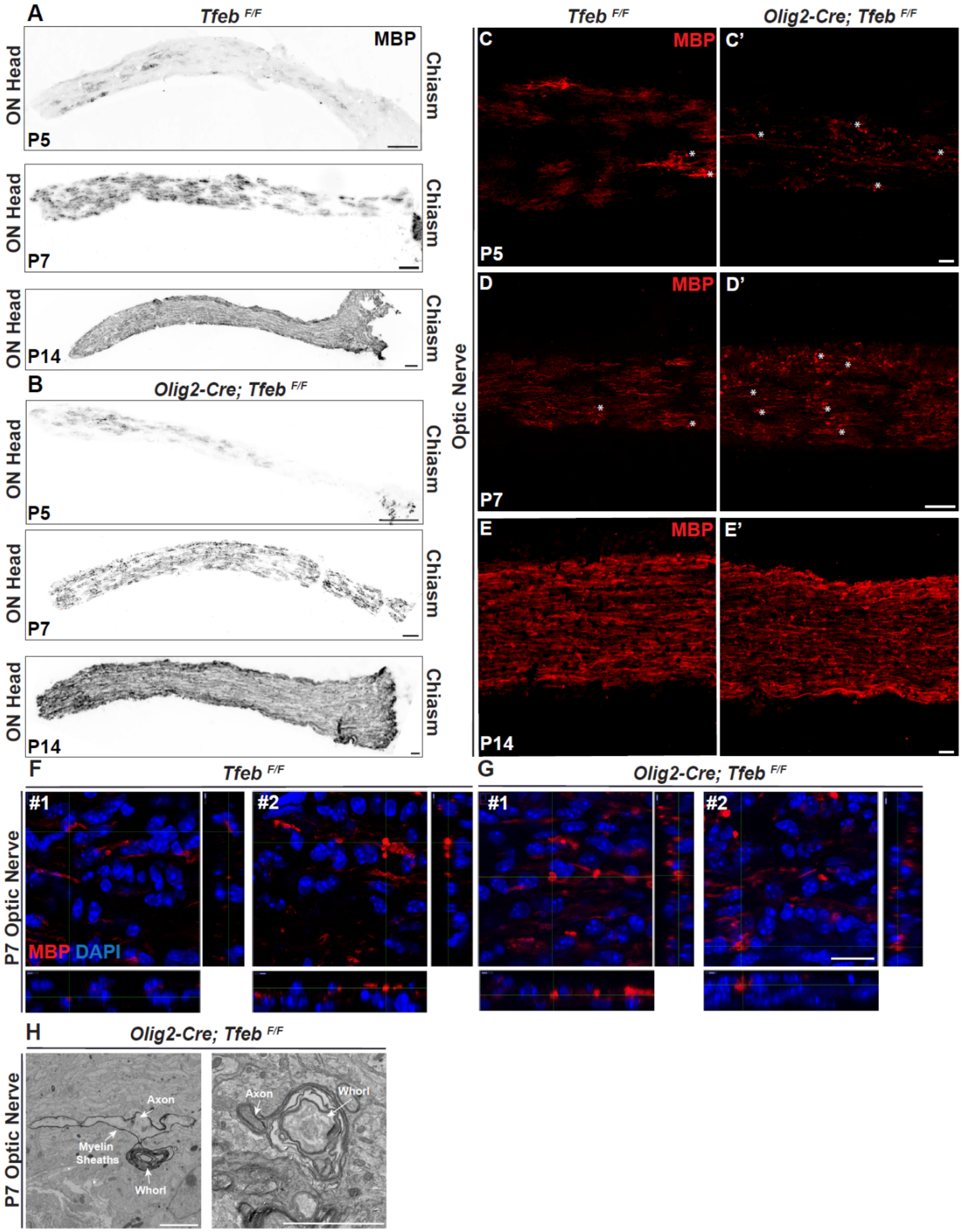


**Fig. S4. MBP+ protrusions emerge at the early onset of axon ensheathment, and early formed whorls are associated with axons.**

**(A and B)** Representative longitudinal sections from *Tfeb*$^{F/F}$ (A) and *Olig2-Cre; Tfeb*$^{F/F}$ (B) optic nerves at P5, P7, and P14, stained with an MBP antibody. Left: optic nerve (ON) head; Right: optic chiasm.

**(C–E')** Representative confocal micrographs from *Tfeb*$^{F/F}$ (C, D, and E) and *Olig2-Cre; Tfeb*$^{F/F}$ (C', D', and E') optic nerves at P5 (C and C'), P7 (D and D'), and P14 (E and E'), stained with an MBP antibody. White asterisks indicate MBP$^{+}$ protrusions.
**(F and G)** Orthogonal views of single confocal planes showing the spatial relationship between MBP$^{+}$ protrusions and DAPI$^{+}$ nuclei in P7 *Tfeb*$^{F/F}$ (F) and *Olig2-Cre; Tfeb*$^{F/F}$ optic nerves (G). (F) and (G) represent the MBP$^{+}$ protrusions shown in Figures 3D and 3E, respectively.
**(H)** Representative TEM micrographs of *Olig2-Cre; Tfeb*$^{F/F}$ optic nerves at P7, showing the connections between whorls and axons (H).
Scale bars: 100 μm for (A) and (B); 20 μm in (C') for (C) and (C'); 20 μm in (D') for (D) and (D'); 20 μm in (E') for (E) and (E'); 20 μm in #2 of (G) for (F) and (G); and 4 μm in (H).

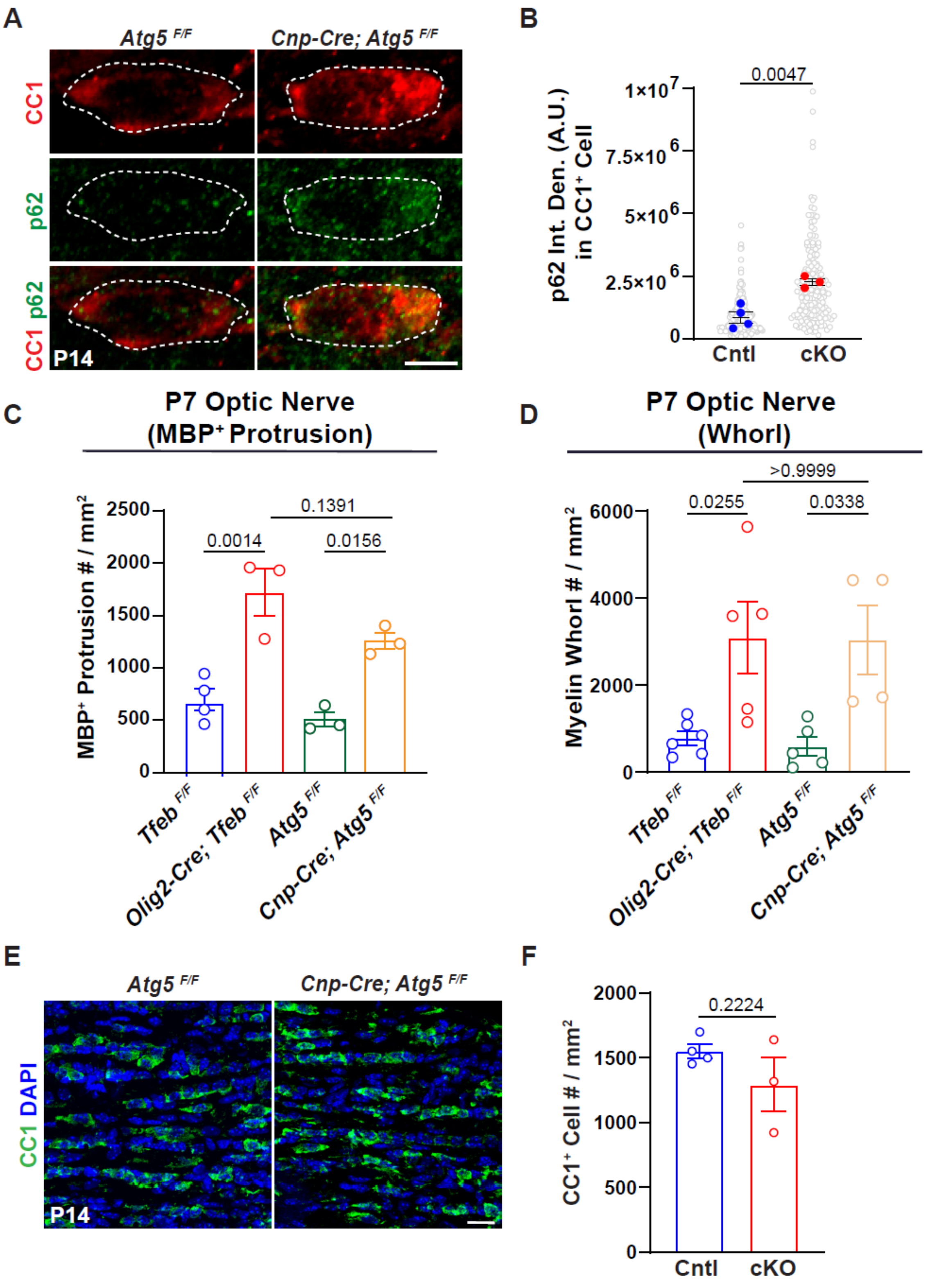
A
Atg5 F/F
Cnp-Cre; Atg5 F/F
CC1
p62
CC1 p62
P14
B
p62 Int. Den. (A.U.) in CC1+ Cell
0.0047
Cntl
cKO
C
P7 Optic Nerve (MBP+ Protrusion)
MBP+ Protrusion # / mm2
0.0014
0.1391
0.0156
Tfeb F/F
Olig2-Cre; Tfeb F/F
Atg5 F/F
Cnp-Cre; Atg5 F/F
D
P7 Optic Nerve (Whorl)
Myelin Whorl # / mm2
>0.9999
0.0255
0.0338
Tfeb F/F
Olig2-Cre; Tfeb F/F
Atg5 F/F
Cnp-Cre; Atg5 F/F
E
Atg5 F/F
Cnp-Cre; Atg5 F/F
CC1 DAPI
P14
F
CC1+ Cell # / mm2
0.2224
Cntl
cKO

**Fig. S5. Characterization of autophagic flux, myelin whorl density, and oligodendrocyte number in *Atg5* conditional knockout optic nerves.**
**(A)** Double immunofluorescence staining against CC1 and p62 in $Atg5^{F/F}$ (left) and *Cnp-Cre;* $Atg5^{F/F}$ (right) optic nerves at P14. Dashed lines demarcate $CC1^+$ oligodendrocyte cell bodies.
**(B)** Quantification of integrated intensity (arbitrary units, A.U.) of p62 immunofluorescence signals in $CC1^+$ cells in P14 $Atg5^{F/F}$ (Cntl) and *Cnp-Cre;* $Atg5^{F/F}$ (cKO) optic nerves. Blue and red circles indicate individual animals, and gray circles indicate individual $CC1^+$ cells used for quantification of p62 immunofluorescence signals. $n$ = 4 $Atg5^{F/F}$ mice, and $n$ = 3 *Cnp-Cre;* $Atg5^{F/F}$ mice.
**(C)** Comparison of $MBP^+$ protrusion densities among $Tfeb^{F/F}$, *Olig2-Cre;* $Tfeb^{F/F}$, $Atg5^{F/F}$, and *Cnp-Cre;* $Atg5^{F/F}$ optic nerves at P7, based on the data shown in Figures 3F and 5F.
**(D)** Comparison of myelin whorl densities among $Tfeb^{F/F}$, *Olig2-Cre;* $Tfeb^{F/F}$, $Atg5^{F/F}$, and *Cnp-Cre;* $Atg5^{F/F}$ optic nerves at P7, based on the data shown in Figures 3H and 6B.
**(E)** Representative confocal micrographs of P14 $Atg5^{F/F}$ and *Cnp-Cre;* $Atg5^{F/F}$ optic nerve sections stained with a CC1 antibody.
**(F)** Quantification of $CC1^+$ cell density in P14 $Atg5^{F/F}$ (Cntl) and *Cnp-Cre;* $Atg5^{F/F}$ (cKO) optic nerves.
Error bars indicate SEM. Two-tailed Student's *t*-test for (B) and (F). One-way ANOVA with Tukey's multiple comparisons test for (C) and (D). Scale bars: 5 μm in (A) and 20 μm in (E).

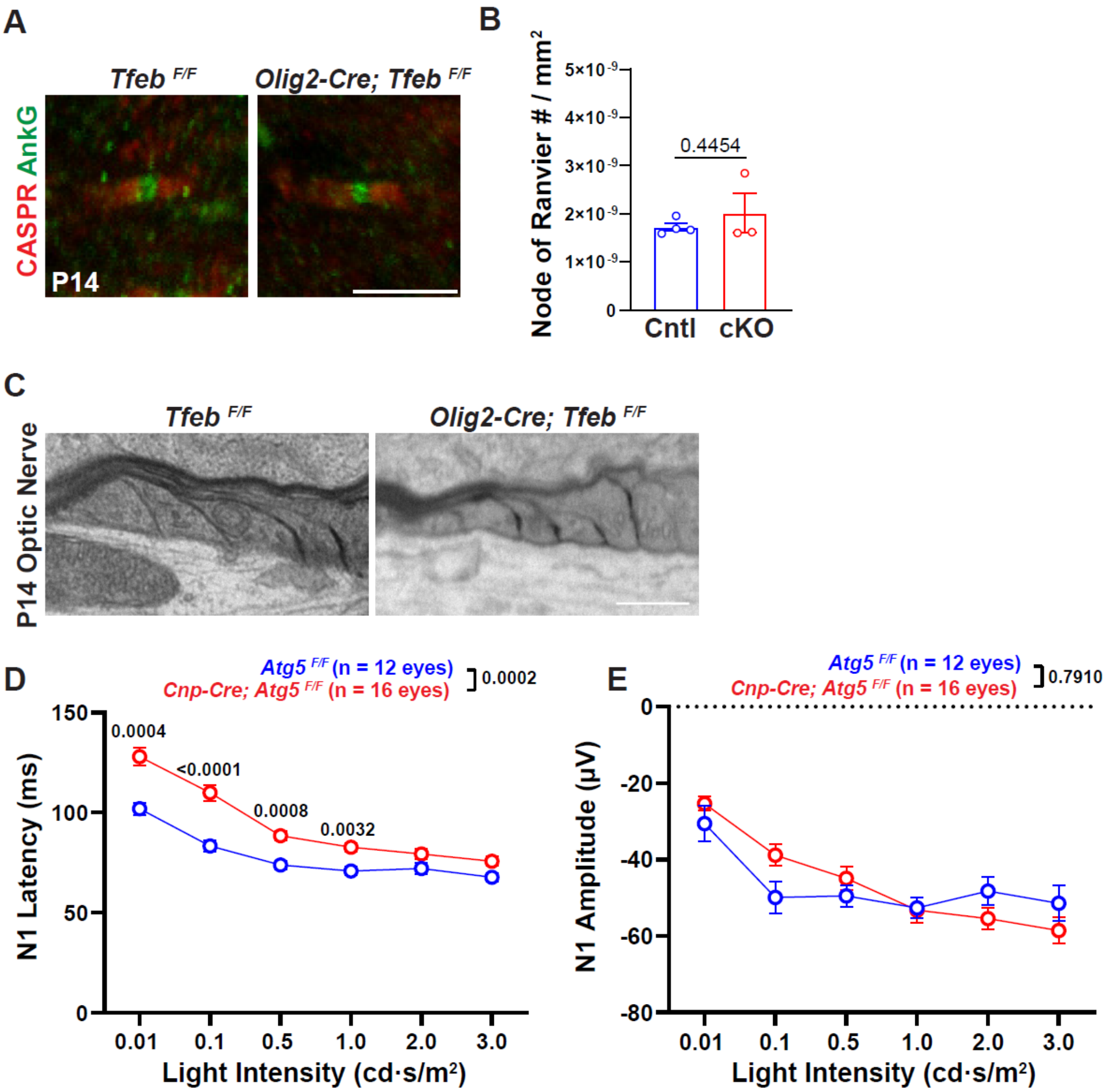


**Fig. S6. Characterization of nodes of Ranvier (NoR) in *Tfeb* cKO optic nerves and VEPs in *Atg5* conditional knockout mice.**

**(A)** Representative micrographs of *Tfeb*$^{F/F}$ and *Olig2-Cre; Tfeb*$^{F/F}$ optic nerve sections at P14, stained with CASPR and AnkG antibodies.

**(B)** Quantification of CASPR$^{+}$ AnkG$^{+}$ NoR density in *Tfeb*$^{F/F}$ (Cntl) and *Olig2-Cre; Tfeb*$^{F/F}$ (cKO) optic nerve sections at P14.

**(C)** Representative longitudinal TEM micrographs of *Tfeb*$^{F/F}$ and *Olig2-Cre; Tfeb*$^{F/F}$ optic nerves at P14, showing the ultrastructure of paranodes.

**(D and E)** VEP N1 latencies (D) and amplitudes (E) in P14 *Atg5*$^{F/F}$ and *Cnp-Cre; Atg5*$^{F/F}$ mice across 0.01, 0.1, 0.5, 1.0, 2.0, and 3.0 cd·s/m$^2$ light intensities. $n$ = 12 *Atg5*$^{F/F}$ eyes. $n$ = 16 *Cnp-Cre; Atg5*$^{F/F}$ eyes.

Error bars indicate SEM. Open circles in (B) represent individual animals. Open circles in (D) and (E) represent the mean value. Two-tailed Student's *t*-test for (B). Two-way ANOVA with Sidak's multiple comparisons test for (D) and (E). Scale bars: 5 µm in (A) and 0.5 µm in (C).

**Movie S1 (separate file).** A video showing 767 consecutive SBEM sections from a P14 *Tfeb*$^{F/F}$ optic nerve, with myelin whorls annotated by different colors. The video is played at 7 SBEM sections per second. Scale bar: 10 μm.

**Movie S2 (separate file).** A 3D rendering video showing annotated whorls from a P14 *Tfeb*$^{F/F}$ optic nerve. Scale bar: 10 μm.

**Movie S3 (separate file).** A video showing 473 consecutive SBEM sections from a P14 *Olig2-Cre; Tfeb*$^{F/F}$ optic nerve, with whorls annotated by different colors. The video is played at 7 SBEM sections per second. Scale bar: 10 μm.

**Movie S4 (separate file).** A 3D rendering video showing annotated whorls from a P14 *Olig2-Cre; Tfeb*$^{F/F}$ optic nerve. Scale bar: 10 μm.

**Movie S5 (separate file).** Consecutive SBEM sections and annotation of a "detached" whorl in a P14 *Tfeb*$^{F/F}$ optic nerve. The video is played at 4 SBEM sections per second. Scale bar: 1 μm.

**Movie S6 (separate file).** Consecutive SBEM sections and annotation of a whorl associated with a myelinated axon in a P14 *Tfeb*$^{F/F}$ optic nerve. The video is played at 4 SBEM sections per second. Scale bar: 1 μm.

**Movie S7 (separate file).** Consecutive SBEM sections and annotation of a "detached" whorl in a P14 *Olig2-Cre; Tfeb*$^{F/F}$ optic nerve. The video is played at 4 SBEM sections per second. Scale bar: 1 μm.

**Movie S8 (separate file).** Consecutive SBEM sections and annotation of a whorl associated with a myelinated axon in a P14 *Olig2-Cre; Tfeb*$^{F/F}$ optic nerve. The video is played at 4 SBEM sections per second. Scale bar: 1 μm.

**Movie S9 (separate file).** 3D rendering of $MBP^+$ processes in P7 *Tfeb*$^{F/F}$ optic nerve, showing the smooth $MBP^+$ internode. See also Figure 3D.

**Movie S10 (separate file).** 3D rendering of $MBP^+$ processes in P7 *Tfeb*$^{F/F}$ optic nerve, showing several $MBP^+$ protrusions along the internode. See also Figure 3D.

**Movie S11 (separate file).** 3D rendering of $MBP^+$ processes in P7 *Tfeb*$^{F/F}$ optic nerve, showing an $MBP^+$ rosette structure formed along the internode. See also Figure 3D.

## SI Reference


1. A. Boey, V. Rybakin, D. Kalicharan, K. Vints, N. V. Gounko, Gold-substituted Silver-intensified Peroxidase Immunolabeling for FIB-SEM Imaging. *J Histochem Cytochem* **67**, 351-360 (2019).
